# Genome mining and UHPLC–QTOF–MS/MS to identify the potential antimicrobial compounds and determine the specificity of biosynthetic gene clusters in *Bacillus subtilis* NCD-2

**DOI:** 10.1186/s12864-020-07160-2

**Published:** 2020-11-05

**Authors:** Zhenhe Su, Xiuye Chen, Xiaomeng Liu, Qinggang Guo, Shezeng Li, Xiuyun Lu, Xiaoyun Zhang, Peipei Wang, Lihong Dong, Weisong Zhao, Ping Ma

**Affiliations:** Institute of Plant Protection, Hebei Academy of Agricultural and Forestry Sciences, Integrated Pest Management Center of Hebei Province, Key Laboratory of IPM on Crops in Northern Region of North China, Ministry of Agriculture and Rural Affairs of China, 437# Dongguan street, Baoding city, 071000 Hebei Province China

**Keywords:** *Bacillus subtilis* NCD-2, Genome mining, UHPLC–QTOF–MS/MS, Secondary metabolites, Fengycin

## Abstract

**Background:**

*Bacillus subtilis* strain NCD-2 is an excellent biocontrol agent against plant soil-borne diseases and shows broad-spectrum antifungal activities. This study aimed to explore some secondary metabolite biosynthetic gene clusters and related antimicrobial compounds in strain NCD-2. An integrative approach combining genome mining and structural identification technologies using ultra-high-performance liquid chromatography coupled to quadrupole time-of-flight tandem mass spectrometry (UHPLC-MS/MS), was adopted to interpret the chemical origins of metabolites with significant biological activities.

**Results:**

Genome mining revealed nine gene clusters encoding secondary metabolites with predicted functions, including fengycin, surfactin, bacillaene, subtilosin, bacillibactin, bacilysin and three unknown products. Fengycin, surfactin, bacillaene and bacillibactin were successfully detected from the fermentation broth of strain NCD-2 by UHPLC-QTOF-MS/MS. The biosynthetic gene clusters of bacillaene, subtilosin, bacillibactin, and bacilysin showed 100% amino acid sequence identities with those in *B. velezensis* strain FZB42, whereas the identities of the surfactin and fengycin gene clusters were only 83 and 92%, respectively. Further comparison revealed that strain NCD-2 had lost the *fenC* and *fenD* genes in the fengycin biosynthetic operon. The biosynthetic enzyme-related gene *srfAB* for surfactin was divided into two parts. Bioinformatics analysis suggested that FenE in strain NCD-2 had a similar function to FenE and FenC in strain FZB42, and that FenA in strain NCD-2 had a similar function to FenA and FenD in strain FZB42. Five different kinds of fengycins, with 26 homologs, and surfactin, with 4 homologs, were detected from strain NCD-2. To the best of our knowledge, this is the first report of a non-typical gene cluster related to fengycin synthesis.

**Conclusions:**

Our study revealed a number of gene clusters encoding antimicrobial compounds in the genome of strain NCD-2, including a fengycin synthetic gene cluster that might be unique by using genome mining and UHPLC–QTOF–MS/MS. The production of fengycin, surfactin, bacillaene and bacillibactin might explain the biological activities of strain NCD-2.

**Supplementary Information:**

The online version contains supplementary material available at 10.1186/s12864-020-07160-2.

## Background

The *Bacillus* genus has received considerable attention as a biological resource for the development of microbial pesticides, partly because some or most of its members form stress-resistant spores that do not harm the environment and are useful in pesticide production [1–3]. *Bacillus subtilis* and its closely related species are ubiquitous inhabitants of soil, and are widely recognized as powerful biocontrol agents against plant soil-borne diseases [4]. The mechanisms used by *B. subtilis* to suppress plant soil-borne diseases include competing with phytopathogens for nutrients and spatial sites, inducing systemic resistance in plants, and inhibiting pathogen growth by producing antimicrobial compounds [5]. The latter is a general characteristic of *B. subtilis* biocontrol agents and plays an important role in the suppression of plant diseases [6, 7]. *B. subtilis* produces more than two dozen antimicrobial compounds with amazing structural variety. Based on their biosynthetic pathways, the antimicrobial compounds are divided into small molecular compounds synthesized by the ribosomal pathway, such as bacteriocins, and peptide compounds synthesized by the non-ribosomal pathway, such as lipopeptides and polyketides [8]. Most antimicrobial compounds are secondary metabolites produced by biocontrol of *Bacillus* spp., and are not necessary for their growth and reproduction but lead to shifts of rhizospheric microbial functional subsystems and affect the availability of nutrients for the plant [9]. Secondary metabolites also function as essential chemical signals for the induction of cellular differentiation in the producing organism and for controlling its metabolism [10, 11].

The genes encoding the secondary metabolites commonly exist in clusters and encode enzyme complexes with multiple functions [12]. The polyketide synthase/non-ribosomal peptide synthetase (PKS/NRPS) gene clusters have been well studied. The PKS pathway polyketides require at least three domains: an acyl transferase, a ketosynthase, and an acyl carrier protein [13]. The NRPS synthetic pathways share a common multicarrier thiotemplate mechanism requiring the cooperation of three basic domains [14]. The adenylation domain selects the cognate amino acid and generates an enzymatically stabilized aminoacyl adenylate. The peptidyl carrier domain is equipped with a 4′-phosphopantetheine prosthetic group, to which the adenylated amino acid substrate is transferred and bonded by a thioester bond. The condensation domain catalyzes the formation of a new peptide bond [13]. The carbon skeleton of the metabolite is synthesized by the core PKS and NRPS enzymes, and is then modified to form the final product with the assistance of various modifying enzymes [15]. The bioactive secondary metabolites produced by the PKS/NRPS pathway in *B. subtilis* have received extensive studies, such as bacilysin [16], bacilysocin [17], surfactin [18], iturin A [19], fengycin [20], mycosubtilin [21], bacillomycins [8], and difficidin [16].

The conventional method for screening new active products is generally based on biological tests, which is time-consuming and sometimes result in repeatedly screened out the same products [22]. Thus, a more rapid and effective screening strategy to detect secondary metabolites was required [23, 24]. Genome mining is a technology that uses modern bioinformatics to recognize specific functional genes or gene clusters from genome sequences [25]. With the rapid development of DNA sequencing technology and the decrease of sequencing cost, a large number of microbial genome sequences have been determined [26], which makes genome mining an accurate and efficient strategy to find new metabolites [25].

*B. subtilis* strain NCD-2 is a plant soil-borne disease-suppressive agent producing lipopeptides, fengycin, and surfactin [27]. Fengycin shows strong antifungal activity, and surfactin facilitates the root colonization. Both fengycin and surfactin play important roles in suppression of plant soil-borne diseases by strain NCD-2 [28]. The purpose of this study was to identify potential secondary metabolites in strain NCD-2, reveal the different gene clusters of the secondary metabolites between strain NCD-2 and the reference strain *B. velezensis* FZB42, and identify the potential secondary metabolites produced by strain NCD-2.

## Results

### Genomic features of strain NCD-2

**Fig. 1:**
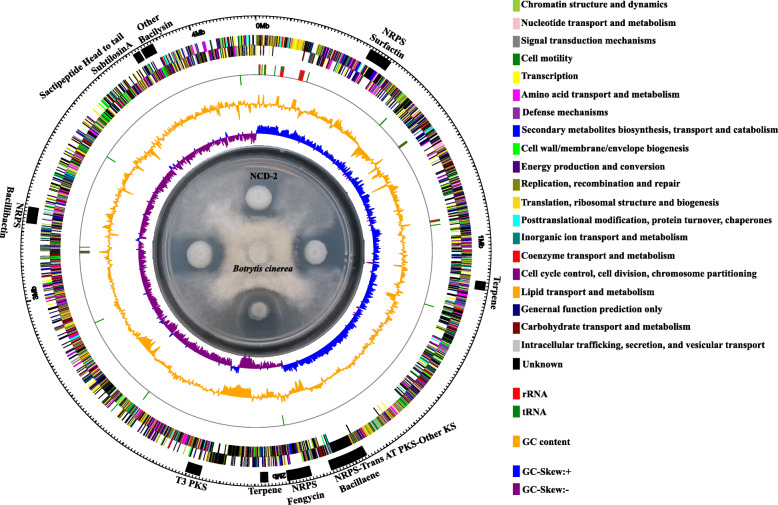
Circular genome of strain NCD-2 with specific features. The circular genome map was created using Circos v0.66 with COG (Cluster of Orthologous Groups of proteins) function annotation. From outside to inside: circle 1, the size of the complete genome; circles 2 and 3, the predicted protein-coding genes on the + and - strands, respectively, where different colours represent different COG function classifications; circle 4, tRNA (green) and rRNA (red); circle 5, G + C content, where peaks outside/inside the circle indicate above or below average GC content, respectively; the inner circle, G + C skew, with G% < C% in purple and with G% > C% in blue. The potato dextrose agar plate inside the representation of the circular genome shows the antifungal activity of strain NCD-2 and its derived strain constructed by atmospheric and room temperature plasma (ARTP) against *Botrytis cinerea*. The black bars outside the circular genome indicate the secondary metabolite biosynthetic gene clusters

A total of 501,671,500 paired-end reads and 5,016,715 clean single reads (412-bp library; paired-ends of 75 bp) were assembled using the software package Velvet [29]. The genome of *B. subtilis* NCD-2 contains 189 contigs (> 133 bp; N90, 16,187) of 4,644,322 bp, with an average G + C content of 43.5%. The final assembled genome comprises 4444 genes, including 4329 protein-coding genes (418 signal peptide-coding genes), 83 tRNA genes for all 20 amino acids, 30 rRNA genes, and 2 clustered regularly interspaced short palindromic repeats (CRISPR) genes. A total of nine putative gene clusters responsible for antimicrobial metabolite biosynthesis were identified. These gene clusters included PKS and NRPS genes (Fig. 1).

### The taxonomic status of strain NCD-2

**Fig. 2:**
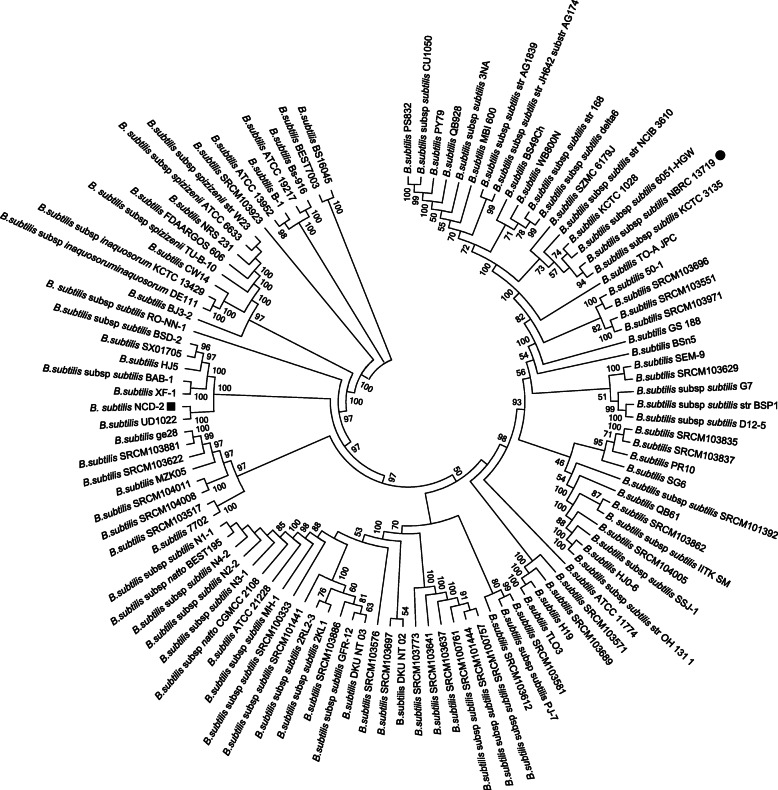
Phylogenetic tree of 113 *B. subtilis* strains based on whole genome alignments. The position of strain NCD-2 in the phylogenetic tree is indicated by a black square mark, and the position of the reference strain *B. subtilis* NBRC 13719 is indicated by a black circle mark. Single Nucleotide Polymorphisms (SNPs) and short insertions or deletions (indels) within the multiple sequence alignments constructed by the REALPHY pipeline were extracted for subsequent phylogeny reconstruction. The phylogenetic tree was constructed using MEGA 5.0 by the Neighbor-joining method, with a bootstrap of 1000 replications. Bootstrap confidence levels > 50% are indicated at the internodes

At present, 272 *B. subtilis* genome sequences have been deposited in the GenBank database, including 113 whole- and 159 incomplete genome sequences. The genome sizes of the 272 *B. subtilis* strains range from 2.68 Mb to 5.35 Mb, and the GC contents range from 42.9 to 46.6%. These genome sequences were downloaded from the GenBank database, and their accession numbers were listed in Additional file 1, Table S1. To analyze the evolution of different *B. subtilis* strains, a phylogenetic tree was constructed based on the complete genome sequences. The 272 strains of *B. subtilis* were divided into four subspecies, *subtilis*, *inaquosorum*, *spizizenii*, and *stercoris* due to producing different bioactive secondary metabolites [30]. As shown in Fig. 2, strain NCD-2 (represented by the black bar) clustered with *B. subtilis* strain UD1022 and was closely related to *B. subtilis* strains XF-1, BAB-1, HJ5, SX01705, and BSD-2.

### Secondary metabolite biosynthetic gene clusters in strain NCD-2

**Fig. 3:**
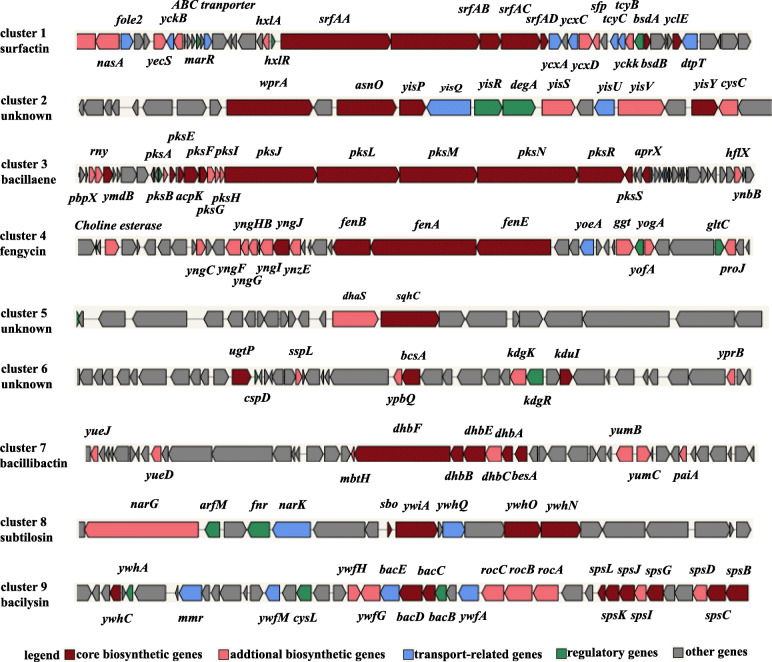
Schematic diagram of nine secondary metabolite biosynthetic gene clusters in *B. subtilis* strain NCD-2. antiSMASH was used to predict potential secondary metabolite biosynthetic gene clusters. Different colour blocks represent genes with different functions; the genes marked with dark red, light red, blue, green, and gray are core biosynthetic, additional biosynthetic, transport-related, regulatory, and other genes, respectively

The secondary metabolite biosynthetic gene clusters in the genome of strain NCD-2 were predicted using antiSMASH [31]. In total, nine such clusters were identified (Table 1), including three NRPSs, two terpenes, one hybrid NRPS-TransAT PKS-Other KS, one type III polyketide, one sactipeptide-head to tail gene cluster, and a gene cluster with unknown function. The structural compositions of the gene clusters are shown in Fig. 3. These clusters were composed of core biosynthetic, additional biosynthetic, transport-related, regulatory, and other genes. Among them, clusters 3, 7, 8, and 9 had 100% amino acid sequence homologies with known gene clusters that synthesize bacillaene, bacillibactin, subtilosin, and bacilysin, respectively (Table 1). Gene cluster 1 showed 82% amino acid similarity with a surfactin synthetase gene cluster, and gene cluster 4 showed 93% amino acid similarity with a fengycin biosynthetic gene cluster in *B. velezensis* strain FZB42. However, gene clusters 2, 5, and 6 did not match any known gene clusters. Clusters 1 and 4 of strain NCD-2 were further compared with their counterparts in the model strain 168 and *B. subtilis* strains closely related to strain NCD-2 in the phylogenetic tree. The predicted fengycin biosynthetic gene cluster in strain NCD-2 contained three genes, *fenEAB*, while all the other strains contained five genes, *fenCDEAB* (Additional file 1, Fig. S1). SrfAB of surfactin was synthesized via typical transcription and translation of *srfAB* in the 11 strains. However, the same SrfAB was potentially assembled with Gms0366 and Gms0367 and then separately transcribed and translated by *gms0366* and *gms0367* in strain NCD-2 (Additional file 1, Fig. S2). Therefore, we hypothesized that the structures and functions of fengycin and surfactin from strain NCD-2 might be different from those in other *B. subtilis* strains.

**Table 1:** Secondary metabolite gene clusters annotated in *B. subtilis* NCD-2 using antiSMASH

Cluster	Type	From	To	Most similar known cluster	Similarity	MIBiG BGC-ID^a^
cluster 1	NRPS	347,853	413,245	surfactin	82%	BGC0000433_c1
cluster 2	Terpene	1,137,768	1,158,574	–	–	–
cluster 3	NRPS-TransAT PKS-Other KS	1,763,940	1,873,766	bacillaene	100%	BGC0001089_c1
cluster 4	NRPS	1,936,035	2,004,508	fengycin	93%	BGC0001095_c1
cluster 5	Terpene	2,060,609	208,250	–	–	–
cluster 6	T3PKS	2,261,562	2,302,659	–	–	–
cluster 7	NRPS	3,225,454	3,275,189	bacillibactin	100%	BGC0000309_c1
cluster 8	Sactipeptide-head to tail	3,817,363	3,838,974	subtilosin	100%	BGC0000602_c1
cluster 9	Other	3,842,273	3,883,691	bacilysin	100%	BGC0001184_c1

^a^Identification numbers of the most similar gene clusters from *B. velezensis* FZB42 provided by the MIBiG BGC database. *NRPS* non-ribosomal peptide synthetase, *PKS* polyketide synthase, *T3PKS* type III polyketide, NRPS-TransAT PKS-Other KS, non-ribosomal peptide synthetase-trans-AT polyketide synthase-Other types of polyketide synthase cluster; Sactipeptide-head to tail, head-to-tail cyclised peptide

### Specificity of the surfactin and fengycin synthetase gene clusters in *B. subtilis* NCD-2

**Fig. 4:**
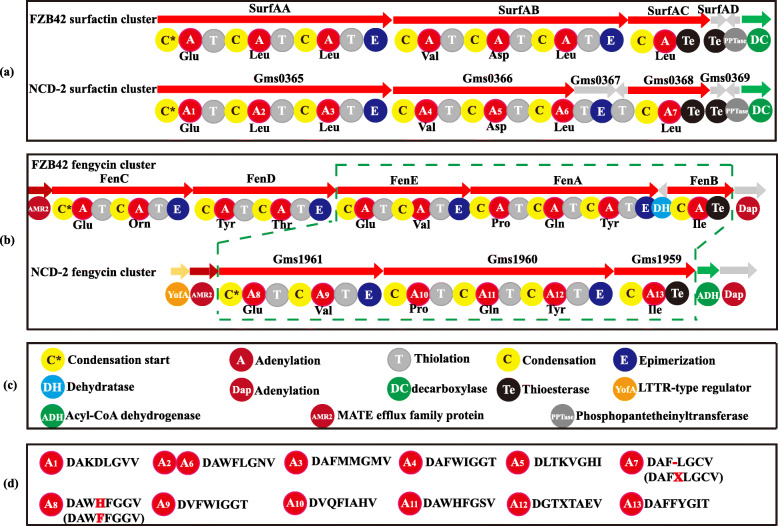
Comparisons of functional domains of core genes involved in synthesizing surfactin and fengycin in NCD-2. The functional domains of the core genes of clusters 1 (**a**) and 3 (**b**) in *B. subtilis* NCD-2. (**c**) The abbreviations indicate the functions of the corresponding structural domains. (**d**) The conserved binding pockets for substrates formed by amino acids in different adenylation domains

The surfactin biosynthetic gene cluster *gms0365–0368* in strain NCD-2 was analyzed using PRISM, and the core genes were selected for PKS/NRPS analysis. Gms0365 had an identical conserved domain, CATCATCATe, with SrfAA in strain FZB42, in which C, A, T, and Te represent the condensation, adenylation, thiolation, and thioesterase domains, respectively (Fig. 4a). Compared with SrfAB in strain FZB42, Gms0366 in strain NCD-2 lacked the T and E domains, but the amino acid residues for the binding pockets in Gms0366 were exactly the same as those of SrfAB. The residues of the different adenylation domains A6 and A2 from the enzymes Gms0365 and Gms0366, respectively, were exactly the same, and both bound the amino acid leucine. Gms0367 only had T and E domains, and no specific substrate-binding domain. The superposition of the Gms0367 and Gms0366 domains formed a complete SrfAB. The T domain was reversed between Gms0367 and Gms0368. Gms0368 contained CATe domains, in which the thioesterase domain releases linear peptide chains. The domains of Gms0368 were the same as those of SrfAC, but the binding pocket (DAF-LGCV) had one missing residue compared with that of strain FZB42 (DAFXLGCV).

The fengycin biosynthetic cluster in strain FZB42 contained five genes *fenCDEAB* (Fig. 4b). However, the same cluster in strain NCD-2 contained only three genes: *gms1961*, *gms1960*, and *gms1959* (Fig. 4b). Gms1961 corresponded to FenE in strain FZB42 had conserved residues of A8 and A9, which bind amino acids Glu and Val, respectively (Fig. 4b). Gms1960 and Gms1959 had conserved amino acid sequences related to FenA and FenB in strain FZB42, respectively. Interestingly, no homologs of FenC and FenD were identified in the genome of strain NCD-2. Consequently, the amino acid sequences of FenC and FenD of strain FZB42 were compared with the strain NCD-2 proteome using BioEdit, and it was found that their most similar proteins were Gms1961 and Gms1960, respectively (Additional file 1, Tables. S2, S3). This finding led to the hypothesis that Gms1961 and Gms1960 performed the functions of FenC and FenD in strain NCD-2, respectively. Thus, Gms1961 and Gms1960 might both have dual functions in the synthesis of fengycin. Gms1961 in strain NCD-2 had the functions of FenE and FenC in strain FZB42, and Gms1960 had the functions of FenA and FenD. However, it should be pointed out that the FenD domain in strain NCD-2 varied greatly with that of FZB42, and we cannot rule out the possibility that other enzymes in NCD-2 might have similar functions as FenD.

**Fig. 5:**
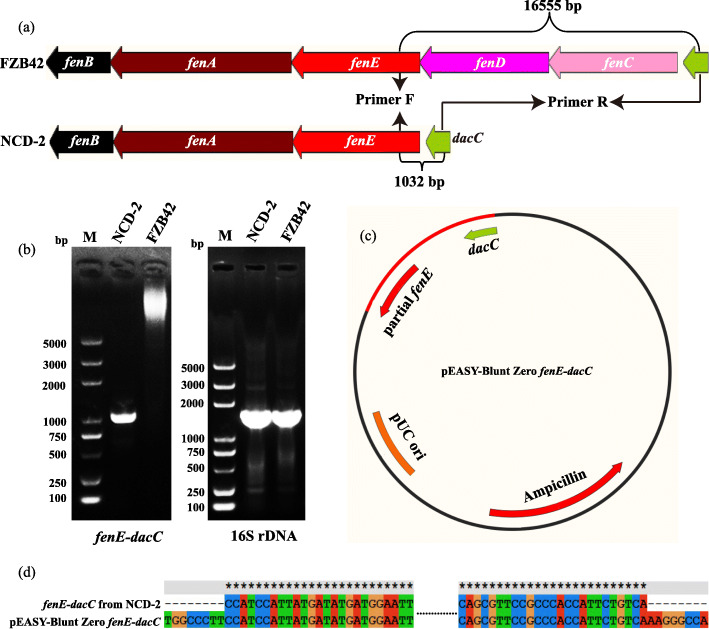
PCR and sequence of the fragment between *fenE* and *dacC*. **a** Schematic diagram used to design primers according to conserved bases from NCD-2 and *B. velezensis* FZB42; **b** PCR of the *fenE-dacC* fragment using the genomic templates NCD-2 and FZB42, with 16S rDNA as an internal reference control; **c** Schematic diagram of the constructed sequencing vector by ligating the *fenE-dacC* fragment to the pEASY-Blunt Zero vector, **d** BLAST of the *fenE-dacC* fragments from NCD-2 and pEASY-Blunt Zero *fenE-dacC*, in which the two sequences of *fenE-dacC* were complete same

**Fig. 6:**
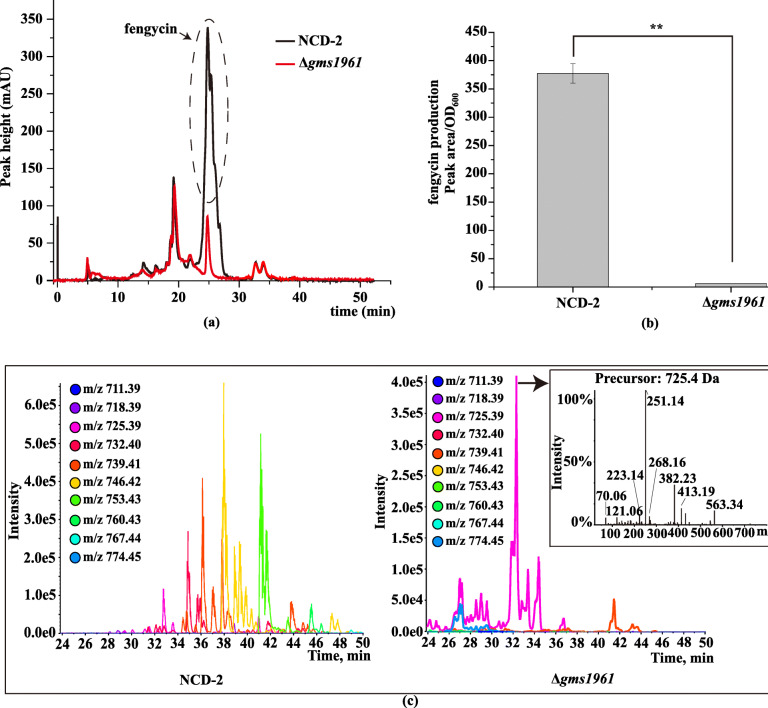
The role of *gms1961* in synthesizing fengycin. **a** FPLC of the lipopeptides of strain NCD-2 and Δ*gms1961*, **b** quantitative production of fengycin in strain NCD-2 and Δ*gms1961*, where the error bars represent the standard deviation and asterisks depict significant differences as measured by the t-test (**p < 0.01), **c** Extract Ions Using Dialog (XIC) and UHPLC-QTOF-MS of fengycin from NCD-2 and Δ*gms1961*. The lipopeptide fengycin exhibited a difference at 25–50 min between strains NCD-2 and Δ*gms1961*, and only the precursor related to *m/z* 725.4 was same (the light purple line), but the fragments were absolutely different from those of fengycin from strain NCD-2

PCR amplification using the primer set targeting the *fenE* and *dacC* genes produced the expected 1032 bp fragment in strain NCD-2 but not in strain FZB42 due to the extremely large size of the target (16,555 bp) (Fig. 5a-b). Sequencing of the 1032 bp fragment and alignment with the gene locus *gms1959–1962* confirmed the lack of *fenC* and *fenD* homologs in this cluster (Fig. 5c-d). Compared to wild-type NCD-2, the in-frame deletion mutant of *gms1961* completely lost fengycin production (Fig. 6a-c).

We further compared the fengycin synthetase gene cluster of NCD-2 with other 11 corresponding clusters from *B. subtilis* strains closely related to strain NCD-2 (Additional file 1, Fig. S1). All the strains contained the fengycin biosynthetic gene cluster *fenCDEAB* (also *ppsABCDE*), except strain NCD-2, which contained *fenEAB*, suggesting that the fengycin biosynthetic gene cluster of strain NCD-2 is unique.

### MS/MS of fengycin and surfactin in NCD-2

**Fig. 7:**
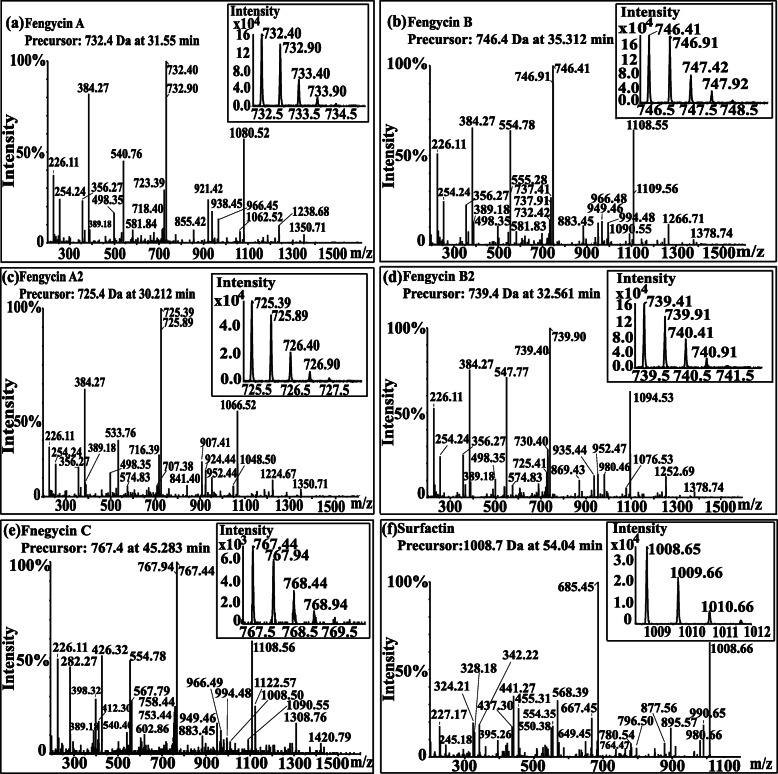
MS/MS spectra of protonated cyclic fengycin and surfactin ions. **a** *m/z* 732.4, **b** *m/z* 746.4, **c** *m/z* 725.4, **d** *m/z* 739.4, **e** *m/z* 767.4, and **f** *m/z* 1008.7

Fengycin was separated from the lipopeptide extract of strain NCD-2 using fast protein liquid chromatography (FPLC) (Additional file 1, Fig. S3). QTOF–MS/MS analysis revealed five fractions in the fengycin cluster (Fig. 7a–e), which had mass-to-charge ratio (*m/z*) values of 732.4, 746.4, 725.4, 739.4, and 767.4 (secondary MS), representing fengycin A, fengycin B, fengycin A2, fengycin B2, and fengycin C, respectively. The typical MS/MS spectra showed the distributions of key fragmentation ions (α and β), representing the linear N-terminal and the cyclic C-terminal segments, respectively, of diverse fengycin species (Fig. 7a–e and Additional file 1, Fig. S4a-b). The MS/MS spectrum of the fengycin ion at *m/z* 732.4 yielded two intense product ions at *m/z* 966.5 and 1080.5, representing fengycin A (Fig. 5a), while the MS/MS spectrum of the fengycin ion at *m/z* 746.4 (Fig. 7b) yielded key product ions at *m/z* 994.5 and 1108.6, representing fengycin B (Fig. 7b). The MS/MS spectrum of the fengycin ion at *m/z* 725.4 yielded two intense product ions at *m/z* 952.4 and 1066.5, representing fengycin A2 (Fig. 7c), while the MS/MS spectrum of the fengycin ion at *m/z* 739.4 (Fig. 7d) yielded key product ions at *m/z* 980.5 and 1094.5 representing fengycin B2 (Fig. 7d). The MS/MS spectrum of the fengycin ion at *m/z* 767.4 yielded two intense product ions at *m/z* 994.5/1008.5 and 1108.6/1122.6 representing fengycin C (Fig. 7e). Five classes of fengycins were identified based on the key product ions of β-hydroxy fatty acid (β-OH FA) with chain lengths varying from C12 to C20 (Table 2, Figs. S5–S9). The MS/MS spectrum of the surfactin ion at *m/z* 1008.7 yielded one intense product ion at *m/z* 685.5 (Fig. 7f and Additional file 1, Fig. S4c). Based on this key product ion, one class of compounds was identified: surfactins (*m/z* values of 994.6, 1008.7, 1022.7 and 1036.7) with fatty acid chains varying from C11 to C15 (Fig. S10).

**Table 2:** Fengycin homologs in NCD-2 based on the key product ions of β-OH-FA with different chain lengths

Fengycin family	[M + 2H]^2+^	β-hydroxy fatty acid
fengycin A	718.4, 725.4, 732.4, 739.4, 745.4, 753.4	C14-C19
fengycin B	718.4, 725.4, 732.4, 739.4, 746.4, 753.4, 760.4, 767.4	C12-C19
fengycin A2	718.4, 725.4, 732.4, 739.4	C15-C18
fengycin B2	725.4, 732.4, 739.4, 746.4, 753.4	C14-C18
fengycin C	760.4, 767.4, 774.5	C18-C20

### Detection of other antimicrobial active compounds in NCD-2

**Fig. 8:**
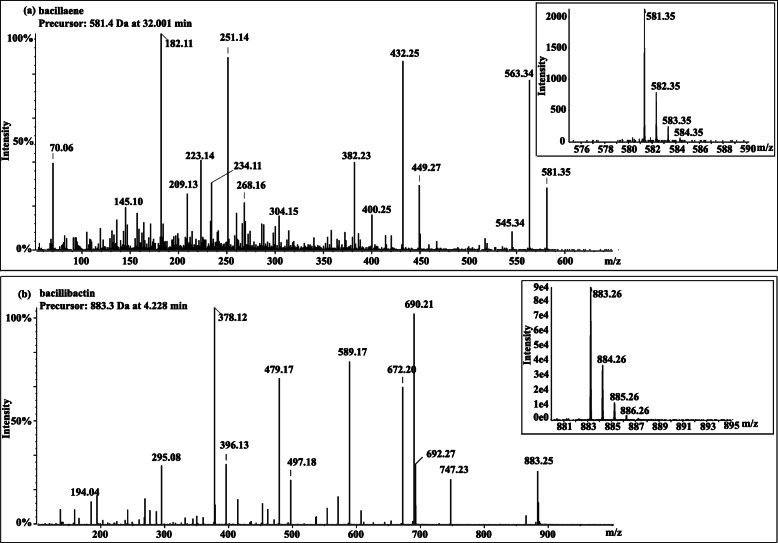
MS/MS spectra of protonated cyclic bacillaene and bacillibactin ions. **a** *m/z* 581.4, **b** *m/z* 883.3

Besides the fengycin and surfactin, other four antimicrobial compounds bacillaene, bacilysin, bacillibactin and subtilosin were also extracted from the fermentation broth of strain NCD-2 by using different extracting methods, respectively. However, only bacillaene and bacillibactin were detectable from the extracts by UHPLC-QTOF-MS (Fig. 8a, b).

## Discussion

*B. subtilis* has the potential to produce two dozen antimicrobial substances, and 5–8% of the *B. subtilis* genome contributes to the production of antimicrobial substances [32]. Some of these substances inhibit the growth of pathogens and the germination of spores. The lipopeptide mixture of *B. subtilis* C232 inhibits the formation of *Verticillium dahliae* microsclerotia [33], and the volatile compounds secreted by *B. subtilis* JA inhibit the conidial formation and mycelial growth of *Glomus etunicatum* [34].

However, certain antimicrobial compounds are synthesized only in response to external stimulation or under special conditions, this made it difficult to harvest all antimicrobial compounds produced by a given *Bacillus* strain using traditional cultivation and extraction methods [22]. Genome mining allows the prediction of metabolites based on genome sequences, including both identified antimicrobial compounds and novel antibiotics that have not been previously described. For example, the new NRPS antibiotic coelichelin was identified by genomic analysis from *Streptomyces coelicolor* [35]. Pseudomycoicidin in *Bacillus pseudomycoides* DSM 12442 was discovered by genome mining and through heterologous expression of its BGC in *Escherichia coli* [36].

Several lipopeptide antibiotics, including fengycin and surfactin have been identified in *B. subtilis* NCD-2 by using traditional cultivation and extraction methods [28]. Fengycin showed strong antifungal abilities against *V. dahliae* and *B. cinerea*. In this study, seven additional secondary metabolite gene clusters were found by genome mining, and some of them were identified using MS/MS. In total, *B. subtilis* NCD-2 had the potential to produce at least 9 kinds of secondary metabolites including surfactin, bacillaene, fengycin, bacillibactin, subtilosin, bacilysin, two terpenes, and one unknown product. Surfactin exhibits antibacterial, antiviral, antitumor and hemolytic action [37]. Bacillaene inhibits bacterial growth by inhibiting prokaryotic protein synthesis [38]. Fengycin shows specific antifungal activity against filamentous fungi [39]. Bacillibactin functions as a siderophore to compete for irons with environmental microbes especially under the iron deficiency conditions. *B. subtilis* expresses genes involved in the synthesis for bacillibactin to pirate other microbial iron [40].. Subtilosin possesses antibacterial activity against a diverse range of bacteria [41]. Bacilysin exhibits antimicrobial activities against both bacteria and *Candida albicans* [42]. However, only fengycin, surfactin, bacillaene and bacillibactin were successfully detected from the extract of strain NCD-2 by UHPLC-MS/MS (Figs. 7, 8). Bacilysin and subtilosin remained undetectable. A likely reason for their undetectability is the low expression level of their biosynthetic gene clusters under the experimental conditions.

*B. velezensis* FZB42 is a model strain of plant beneficial rhizobacteria. Thirteen gene clusters involved in the non-ribosomal and ribosomal synthesis of secondary metabolites with putative antimicrobial action have been identified within the genome of strain FZB42, including fengycin. The mechanism of fengycin synthesis has been well studied in *B. velezensis* strain FZB42 [43]. *B. subtilis* 168 has the entire gene cluster for synthesizing fengycin, but it can not produce fengycin because of a deficiency of a native *sfp* gene [44]. The BGC repository MIBiG (Minimum Information about a Biosynthetic Gene cluster) includes only has one fengycin biosynthetic gene cluster from *B. velezensis* FZB42 [45, 46]. Therefore, the fengycin biosynthetic gene cluster of strain NCD-2 was compared with that of *B. velezensis* FZB42. Fengycin comprises a peptide ring circled by 10 amino acids with a fatty acid chain tail. The fengycin biosynthetic gene clusters in most producing strains consist of the genes *fenCDEAB* (38 kb), which encode 5 enzymes, among which FenC recognizes and carries glutamate and ornithine; FenD recognizes and carries tyrosine and threonine, FenE recognizes and carries glutamate and valine; FenA recognizes and carries proline, glutamine, and tyrosine; and FenB recognizes and carries isoleucine. FenCDEAB recognizes 10 amino acids and carries them to the β-OH FA chain to form fengycin [47–49]. However, NCD-2 only had *fenEAB*, but no *fenC* and *fenD*, compared with the typical cluster structure of *fenCDEAB* in strain FZB42 strain and other 10 *Bacillus* strains (Fig. 4b and Additional file 1, Fig. S1). To exclude the errors introduced by genome sequencing or assembly, the fragment between *fenE* and *dacC* was cloned and sequenced, and it was confirmed that *fenC* and *fenD* were lost in strain NCD-2 (Fig. 5a-d). To identify the enzymes FenC and FenD in the NCD-2 genome, their amino acid sequences from FZB42 were selected to screen for homologs by scanning the local NCD-2 proteome using BioEdit. The protein Gms1961 in strain NCD-2 had the greatest similarity to FenC at an amino acid sequence level (Additional file 1, Table S2). The Gms1961 protein contained 2550 amino acids, with a molecular weight of 287.50 kDa. The substrate bound by the adenylation domain of the Gms1961 protein was predicted (Additional file 1, Table S4). The adenylation A9 domain bound valine and N5-hydroxyornithine, with the latter being a transitional form of ornithine combined with the adenylation domain [50]. The UHPLC-QTOF-MS/MS results of the fengycins revealed the amino acid ornithine at position 2 in all of the examined structures (Fig. 7a–e), indicating the presence of a protein that transports ornithine in the NCD-2 strain. We thus hypothesized that Gms1961 functions as FenC and FenE. The same analysis was performed using the Gms1960 protein, which had the greatest similarity with FenD (Additional file 1, Table S3). However, the FenD domains in Gms1960 and FZB42 varied greatly. Therefore, it was hypothesized that Gms1960 or other enzymes function similarly to FenD.

Although lacking two important genes, *fenC* and *fenD*, strain NCD-2 was capable of producing 26 homologs of 5 types of fengycins (A, B, A2, B2, and C) (Additional file 1, Fig. S4). The amino acids at positions 6 and 10 in the cyclic peptide ring of fengycin determine its structural type. When the amino acid at position 6 was valine with isoleucine or valine at position 10, fengycin B or fengycin B2, respectively, was produced (Fig. 7a, b and Additional file 1, Fig. S4); however, when the amino acid at position 6 was alanine with isoleucine or valine at position 10, fengycin A or fengycin A2, respectively, was produced (Fig. 7c, d and Additional file 1, Fig. S4). When the amino acid at position 6 was isoleucine or leucine with valine at position 10, fengycin C was produced (Fig. 7e and Additional file 1, Fig. S4). Each fengycin type had different homologs according to the number of carbon atoms in its β-OH FA chain, and the molecular weights of each homologs differed by 14 (−CH2) [51]. Compared to the short-chain varieties, long-chain fatty acids increase the hydrophobic activities of lipopeptides, making them more likely to have membrane-bound antimicrobial effects [52]. A *B. circulans* strain produces four fengycin homologs, but only fengycins with C16 and C17 carbon atoms in their β-OH FA chains have antibacterial activity [53]. Among the 26 fengycin homologs produced by strain NCD-2, 14 fengycins had more than 16 carbon atoms in their β-OH FA chains, which might be the most important composition for antimicrobial function. The *B. siamensis* SCSIO 05746 strain produces a large number of fengycin homologs, including 19 homologs of fengycin B [54]. Using MS/MS analysis, the five fengycins produced by the NCD-2 strain were divided into 26 homologs (Fig. 7a–e and Additional file 1, Fig. S5-S9). Therefore, NCD-2 is currently the strain with the largest number of known fengycin homologs [55].

During microbial synthesis of secondary metabolites, such as lipopeptide, the relatively high energy-consuming process of protein synthesis takes priority [56]. Excessive energy consumption is not conducive to the normal growth of microbes, and, generally, microbes produce antibiotics in large amounts only under stress, such as encountering pathogens [57]. We hypothesized that the essential biosynthetic genes *fenEAB* involved in fengycin synthesis were retained, while another two important biosynthetic genes *fenCD* were lost in the long-term evolution of strain NCD-2. Five fengycins were still produced. Gms1961 might serve the dual roles of FenC and FenE, indicating that NCD-2’s fengycin biosynthetic process is unique to the strain and is more energy-efficient than the process used in the other strains.

## Conclusions

Genome mining and UHPLC–QTOF–MS/MS analysis revealed 9 gene clusters encoding antimicrobial compounds in the genome of *Bacillus subtilis* NCD-2. Among them, the fengycin biosynthetic gene cluster containing *fenEAB* genes is unique to strain NCD-2 compared with the other tested *B. subtilis* strains. Strain NCD-2 might employ a unique mechanism for synthesizing fengycin, which may shed new light on the synthesis and evolution of antimicrobial lipopeptides through the NRPS pathway.

## Methods

### Microorganisms and culture conditions

*B. subtilis* NCD-2 was routinely grown at 37 °C on Luria Bertani medium. For lipopeptide, bacillaene, bacilysin, bacillibactin and subtilosin production, strain NCD-2 was grown in Landy broth [58], PA medium [59], MSA medium [60], and TSB medium [61] at 30 °C and 180 rpm. The phytopathogen *Botrytis cinerea* BC-10 was used for antifungal activity test following the method described by Guo et al. [28] with some modifications. Briefly, a 6-mm diameter disc of *B. cinerea* was placed in the center of a 9-cm potato dextrose agar (PDA) plate, and the plate was inoculated 2 cm from the center with *B. subtilis* NCD-2 using a sterilized toothpick. Finally, the diameter of the inhibition zone was measured after a 3-d incubation at 25 °C.

### Genome sequencing of strain NCD-2

The Illumina Solexa platform (BGI, Shenzhen, China) was used for whole-genome sequencing following the method described by Karim [62] with some modifications. The quality of reads was checked using FastQC (http://www.bioinformatics.babraham.ac.uk/projects/fastqc/) [63], and paired-end reads were trimmed using Sickle (https://github.com/najoshi/sickle), and were assembled using the software package Velvet [29]. QUAST 5.02 was used to assess the quality of contigs and scaffolds [64]. The assembled scaffolds were annotated using Prokka (version v.1.13) [65]. Annotation of the genome of strain NCD-2 was performed using the NCBI Prokaryotic Genomes Automatic Annotation Pipeline (http://www.ncbi.nim.nih.gov/genome/annotation_prok/) utilizing GeneMark, Glimmer, and tRNAscan-SE tools [66], and functional annotation was carried out using the Rapid Annotations by subsystems Technology (RAST) server with the seed database [67]. Finally, the genome of strain NCD-2 was deposited in the National Center for Biotechnology Information (NCBI; https://www.ncbi.nlm.nih.gov/) under the GenBank accession number CP023755.

### Evolutionary analysis, signal peptide and CRISPR repeat detection

Whole-genome sequences of *B. subtilis* and closely related species were downloaded from the NCBI database, and the REALPHY website (http://realphy.unibas.ch) [68] was used to perform genome-wide comparisons with the default parameters. A phylogenetic analysis was conducted using MEGA5 [69] with the Maximum Composite Likelihood parameter model [70]. A phylogenetic tree was constructed using the Neighbor-joining algorithm method with bootstrap values based on 1000 replications. The signal peptide was predicted using the SignalP-5.0 website (www.cbs.dtu.dk/services/SignalP-5.0/) [71]. CRISPR repeats were detected using CRISPRCasFinder (https://crisprcas.i2bc.paris-saclay.fr/CrisprCasFinder/Index) [72].

### Prediction and specificity analysis of secondary metabolite biosynthetic gene clusters

Secondary metabolite biosynthetic gene clusters for strain NCD-2 were detected using antiSMASH (http://antismash.secondarymetabolites.org) [31, 73] and PRISM (http://grid.adapsyn.com/prism/) [74] with the default parameters. The functional domain predictions for PKS/NRPS in the predicted gene clusters were analyzed using the PKS/NRPS Analysis Website (http://nrps.igs.umaryland.edu/) [75]. Typical PKS and NRPS sequences were selected for genomic and proteomic scanning after using BioEdit software to create a local BLAST based on strain NCD-2’s genome and proteome, respectively.

### Detection of FenC and FenD loss in the genome of strain NCD-2

FenC and FenD are two important enzymes for synthesizing fengycin. A pair of degenerate primers targeting *fenE* (5′- CCRTCCATKAYGATATGATG − 3′) and *dacC* (5′- TGACAGAATGRYGGGMGGAAC − 3′) were designed based on the conserved bases of *fenE* and *dacC* in strain NCD-2 and *B. velezensis* strain FZB42. The 16S rDNA (27-F/1492R) primers were used as a positive control [76]. The amplification procedure included a denaturation step at 95 °C for 2 min, followed by 32 cycles of 20 s strand separation at 95 °C, 20 s annealing at 55 °C, and 90 s elongation at 72 °C, and an elongation step of 5 min at 72 °C. The target fragment from NCD-2 was purified by a gel extraction kit (Sangon, Shanghai, China), ligated to blunt-ended vector (Transgen, Beijing, China) and sequenced by BGI company (Shenzhen, China). The fragment sequence was deposited at NCBI with the accession number of MT984302.

### Separation of lipopeptides by FPLC

Lipopeptides were extracted using the method described by Guo et al. [28]. Briefly, strain NCD-2 or derived strain Δ*gms1961* were cultured in 100 mL Landy broth [58] at 30 °C for 72 h with shaking at 180 rpm. The cell-free supernatant was obtained by centrifugation at 8000×g for 30 min at 4 °C. The supernatant was adjusted to pH 2.0 with 6 mol/L HCl and stored for 12 h at 4 °C. After centrifugation at 10,000×g, for 20 min, the resulting pellet was extracted with 10 mL methanol under continuous magnetic stirring for 2 h. The obtained extracts were sterilized by passing through 0.45-μm filters (Millex-GV, Millipore, Billerica, MA, USA) to obtain crude lipopeptides. The crude lipopeptides were separated and purified using an AKTA Purifier (GE Healthcare, Uppsala, Sweden) with a SOURCE 5RPC ST 4.6/150 column as described previously [77]. The lipopeptides were eluted with solvent A [2% acetonitrile containing 0.065% trifluoroacetic acid (TFA) (V/V)] and solvent B [80% acetonitrile containing 0.05% TFA (V/V)] using a linear gradient of 0–100% acetonitrile over 57 min at a flow rate of 1 mL/min. The detection wavelength was 215 nm. All the main peaks were automatically collected by FPLC. Finally, each peak was concentrated using a rotary evaporator and was analyzed using UHPLC-QTOF–MS/MS.

### UHPLC–QTOF–MS/MS

UHPLC–QTOF–MS/MS analysis was conducted on a hybrid quadrupole time-of-flight tandem mass spectrometer (AB SCIEX TripleTOF 5600 Q-TOF/MS, Foster City, CA, USA) with an HPLC (Shimadzu, Kyoto, Japan) equipped with LC-30 AD binary pumps, a SIL-30 AC autosampler, and a CTO-30 AC column oven. A C18 reversed phase LC column (Shim-pack GIST 2-μm particles, 2.1 mm × 100 mm) was used for separation. The mobile phases A and B were water and acetonitrile, respectively, with 0.1% formic acid in both phases and with the optimized linear gradient elution procedure as follows: 0.0–0.5 min, 30% B; 0.5–50 min, 60% B; 50–52 min, 95% B; 52–55 min, 95% B; 55–55.1 min, 30% B; and 55.1–60 min, 30% B. The injection volume was 20 μL with a flow rate of 0.30 mL/min. The column oven was set at 40 °C. MS analysis was performed using a 5600 TripleTOF system equipped with a DuoSpray™ Ion Source, and the data were processed using Analyst TF 1.7 software (Applied Biosystems Sciex, Toronto, ON, Canada). PeakView™ software 2.0 (Applied Biosystems Sciex, Toronto, ON, Canada) was used to investigate and interpret the mass spectral data, with special tools for processing accurate mass data and structural elucidation. The DuoSpray™ ion source was used in positive ion mode. The instrumental parameters were set as follows: ion spray voltage floating, 5000 V; nebulizing gas, 50 psi; heater gas, 50 psi; curtain gas, 35 psi; temperature, 350 °C; declustering potential, 100 V; collision energy, TOF MS experiments: 10.0 V. TOF-MS/MS experiments: rolling collision energy, with collision energy spread 5 V. The data was acquired using Information Dependent Acquisition for a single run analysis with m/z range of 200–2000 in TOF MS and 50–1600 in MS/MS.

### Detection of bacillaene, bacilysin, bacillibactin and subtilosin

For bacillaene, strain NCD-2 was cultured in 100 mL Landy broth at 30 °C for 72 h with shaking at 180 rpm, and bacillaene was extracted with methanol using the method described by Reddick et al. [78]. For bacilysin, strain NCD-2 was cultured in 100 mL PA medium at 30 °C for 72 h with shaking at 180 rpm, and bacilysin was extracted with ice-cold ethanol as described by Wu et al. [59]. For bacillibactin, strain NCD-2 was cultured in 100 mL MSA medium at 30 °C for 72 h, and bacillibactin was extracted with ethanol as described by Li et al. [60]. For subtilosin, strain NCD-2 was cultured in 100 mL TSB medium at 30 °C for 72 h, and subtilosin was extracted with precipitation with 65% ammonium sulphate as described by Charles et al. [61]. The extracts were detected by UHPLC-QTOF-MS/MS as described above.

## Supplementary Information

**Additional file 1 : Fig. S1**. Fengycin biosynthetic gene clusters of different strains that have a close relation with NCD-2 or the model strains. **Fig. S2**. Surfactin biosynthetic gene clusters of different strains that have a close relation with NCD-2 or the model strains. **Fig. S3**. Elution of lipopeptides separated from the crude methanolic extract using an AKTA Purifier. **Fig. S4**. Primary structures of fengycins and surfactins. **Fig. S5**. Fengycin A of a β-OH FA with a chain length varying from C14 to C19 identified based on key product ions. **Fig. S6**. Fengycin B of a β-OH FA with a chain length varying from C12 to C19 identified based on key product ions. **Fig. S7**. Fengycin A2 of a β-OH FA with a chain length varying from C15-C18 identified based on key product ions. **Fig. S8**. Fengycin B2 of a β-OH FA with a chain length varying from C14-C18 identified based on key product ions. **Fig. S9**. Fengycin C of a β-OH FA with a chain length varying from C18-C20 identified based on key product ions. **Fig. S10**. Surfactin of a fatty acid with a chain length varying from C11-C15 identified based on key product ions. **Fig. S11** Original, full-length gel images. **Table S1**. All *B. subtilis* strains with the assembly level of chromosome and their RefSeq assembly accessions. **Table S2**. Homologues of FenC of FZB42 detected by scanning the local NCD-2 proteome in BioEdit. **Table S3**. Homologues of FenD of FZB42 detected by scanning the local NCD-2 proteome in BioEdit. **Table S4**. Adenylation domain binding amino acids predicted by PRISM.

## Data Availability

The datasets used and analyzed in the current study are available from the corresponding author on reasonable request. The genome of strain NCD-2 was deposited at NCBI (https://www.ncbi.nlm.nih.gov) under the GenBank accession number CP023755 (https://www.ncbi.nlm.nih.gov/nuccore/CP023755.1?report=fasta) and the fragment *fenE-dacC* sequence of NCD-2 was deposited at NCBI with the accession number MT984302. All *B. subtilis* strain with the assembly level of complete genome or chromosome could be obtained at NCBI by their RefSeq assembly accession numbers which were listed in Table S1. The genome of *B. velezensis* FZB42 was downloaded at NCBI under the GenBank accession number CP000560.2 (https://www.ncbi.nlm.nih.gov/nuccore/CP000560.2).

## Abbreviations

UHPLC-QTOF-MS/MS: Ultra-high-performance liquid chromatography coupled to quadrupole-time-of-flight tandem mass spectrometry
A domain: Adenylation domain
C domain: Condensation domain
T domain: Thiolation domain
Te: Thioesterase domain
E domain: Epimerization domain
N90: The minimum contig length to cover 90% of the genome
CRISPR: clustered regularly interspaced short palindromic repeats
PDA: Potato dextrose agar
BGC: Biosynthetic gene cluster
FPLC: Fast protein liquid chromatography
*m/z*: mass-to-charge ratio
TFA: Trifluoroacetic acid
β-OHFA: β-hydroxy-fatty acid
