# Supplementary material for: Genome mining and UHPLC–QTOF–MS/MS to identify the potential antimicrobial compounds and determine the specificity of biosynthetic gene clusters in Bacillus subtilis NCD-2
Source: BMC Genomics. 2020 Nov 5;21:767. doi: 10.1186/s12864-020-07160-2 (PMC7643408; doi:10.1186/s12864-020-07160-2)
Supplement: Supplementary file 1 — Additional file 1 : Fig. S1. Fengycin biosynthetic gene clusters of different strains that have a close relation with NCD-2 or the model strains. Fig. S2. Surfactin biosynthetic gene clusters of different strains that have a close relation with NCD-2 or the model strains. Fig. S3. Elution of lipopeptides separated from the crude methanolic extract using an AKTA Purifier. Fig. S4. Primary structures of fengycins and surfactins. Fig. S5. Fengycin A of a β-OH FA with a chain length varying from C14 to C19 identified based on key product ions. Fig. S6. Fengycin B of a β-OH FA with a chain length varying from C12 to C19 identified based on key product ions. Fig. S7. Fengycin A2 of a β-OH FA with a chain length varying from C15-C18 identified based on key product ions. Fig. S8. Fengycin B2 of a β-OH FA with a chain length varying from C14-C18 identified based on key product ions. Fig. S9. Fengycin C of a β-OH FA with a chain length varying from C18-C20 identified based on key product ions. Fig. S10. Surfactin of a fatty acid with a chain length varying from C11-C15 identified based on key product ions. Fig. S11 Original, full-length gel images. Table S1. All B. subtilis strains with the assembly level of chromosome and their RefSeq assembly accessions. Table S2. Homologues of FenC of FZB42 detected by scanning the local NCD-2 proteome in BioEdit. Table S3. Homologues of FenD of FZB42 detected by scanning the local NCD-2 proteome in BioEdit. Table S4. Adenylation domain binding amino acids predicted by PRISM. [file 12864_2020_7160_MOESM1_ESM.zip › Supplementary material.docx]

**Supplementary material**

**Table S1** All *B. subtilis* strain with the assembly level of complete genome or chromosome and their RefSeq assembly accession.

| strain | RefSeq assembly accession | strain | RefSeq assembly accession | strain | RefSeq assembly accession |
| --- | --- | --- | --- | --- | --- |
| 168 | GCF_000155325.1 | SRCM103571 | GCF_004103595.1 | NBRC 13719 | GCF_006741845.1 |
| BEST7003 | GCF_000523045.1 | SRCM103576 | GCF_004119615.1 | RO-NN-1 | GCF_000227485.1 |
| BSn5 | GCF_000186745.1 | SRCM103581 | GCF_004119655.1 | AG1839 | GCF_000699525.1 |
| BS49Ch | GCF_000953615.1 | SRCM103612 | GCF_004119775.1 | BAB-1 | GCF_000349795.1 |
| HJ5 | GCF_000973605.1 | SRCM103622 | GCF_004119835.1 | BSP1 | GCF_000321395.1 |
| KCTC 1028 | GCF_000971925.1 | SRCM103629 | GCF_004119815.1 | AG174 | GCF_000699465.1 |
| PY79 | GCF_000497485.1 | SRCM103637 | GCF_004119875.1 | NCIB 3610 | GCF_000186085.1 |
| QB928 | GCF_000293765.1 | SRCM103641 | GCF_004119555.1 | OH 131.1 | GCF_000706705.1 |
| 50-1 | GCF_003184225.1 | SRCM103689 | GCF_004119535.1 | 2KL1 | GCF_003665395.1 |
| 7702 | GCF_002272405.1 | SRCM103696 | GCF_004119595.1 | 2RL2-3 | GCF_003665275.1 |
| ATCC 11774 | GCF_004101945.1 | SRCM103697 | GCF_004119635.1 | 3NA | GCF_000827065.1 |
| ATCC 13952 | GCF_000772125.1 | SRCM103773 | GCF_004119675.1 | 168G | GCF_001703495.1 |
| ATCC 19217 | GCF_000772165.1 | SRCM103835 | GCF_004119715.1 | BSD-2 | GCF_001465815.1 |
| ATCC 21228 | GCF_002982175.1 | SRCM103837 | GCF_004119695.1 | CU1050 | GCF_001541905.1 |
| B-1 | GCF_000769515.1 | SRCM103862 | GCF_004101345.1 | D12-5 | GCF_001596535.1 |
| BJ3-2 | GCF_002893805.1 | SRCM103881 | GCF_004101445.1 | delta6 | GCF_001660525.1 |
| Bs-916 | GCF_000772205.1 | SRCM103886 | GCF_004101365.1 | G7 | GCF_004328925.1 |
| BS16045 | GCF_001720505.1 | SRCM103923 | GCF_004101405.1 | GFR-12 | GCF_003665195.1 |
| CW14 | GCF_002163815.1 | SRCM103971 | GCF_004101465.1 | IITK SM | GCF_003426125.1 |
| DKU_NT_02 | GCF_002269175.1 | SRCM104005 | GCF_004101425.1 | KCTC 3135 | GCF_001697265.1 |
| DKU_NT_03 | GCF_002269195.1 | SRCM104008 | GCF_004101485.1 | MH-1 | GCF_003665235.1 |
| FDAARGOS_606 | GCF_006364495.1 | SRCM104011 | GCF_004101565.1 | N1-1 | GCF_003665335.1 |
| ge28 | GCF_002202055.1 | SX01705 | GCF_002216085.1 | N2-2 | GCF_003665315.1 |
| GS 188 | GCF_002220075.1 | SZMC 6179J | GCF_001604995.1 | N3-1 | GCF_003665355.1 |
| H19 | GCF_005234095.1 | TLO3 | GCF_002290305.1 | N4-2 | GCF_003665295.1 |
| HJ0-6 | GCF_001704095.1 | TO-A JPC | GCF_001037985.1 | PJ-7 | GCF_003665215.1 |
| MBI 600 | GCF_005160425.1 | UD1022 | GCF_001015095.1 | SRCM100333 | GCF_002201995.1 |
| MZK05 | GCF_003612735.1 | WB800N | GCF_003610955.1 | SRCM100757 | GCF_002173715.1 |
| NRS 231 | GCF_005153965.1 | DE111 | GCF_001534785.1 | SRCM100761 | GCF_002201955.1 |
| PR10 | GCF_005849145.1 | KCTC 13429 | GCF_003148415.1 | SRCM101392 | GCF_002202035.1 |
| PS832 | GCF_000789295.1 | BEST195 | GCF_000209795.2 | SRCM101441 | GCF_002173615.1 |
| QB61 | GCF_003148355.1 | CGMCC 2108 | GCF_001565875.1 | SRCM101444 | GCF_002173695.1 |
| SEM-9 | GCF_006165085.1 | ATCC 6633 | GCF_006094475.1 | SSJ-1 | GCF_003665255.1 |
| SG6 | GCF_000782835.1 | W23 | GCF_000146565.1 | XF-1 | GCF_000338735.1 |
| SRCM103517 | GCF_004103535.1 | TU-B-10 | GCF_000227465.1 | NCD-2 | GCF_002556525.1 |
| SRCM103551 | GCF_004103555.1 | 6051-HGW | GCF_000344745.1 |  |  |

**Table S2** The homologues of FenC of FZB42 detected by scanning the local NCD-2 proteome in BioEdit.

| protein number | score | similarity | E-value | function description |
| --- | --- | --- | --- | --- |
| Gms1961 | 2701 | 55 | 0.0 | FenE |
| Gms1960 | 2036 | 43 | 0.0 | FenA |
| Gms0365 | 1639 | 37 | 0.0 | SrfAA |
| Gms0366 | 1296 | 34 | 0.0 | SrfAB |
| Gms3368 | 1127 | 34 | 0.0 | DhbF |
| Gms1826 | 572 | 27 | e-164 | PKSJ |
| Gms1829 | 516 | 30 | e-147 | PKSN |
| Gms0367 | 489 | 39 | e-138 | Surfactin synthase subunit 2 |
| Gms1959 | 478 | 30 | e-135 | FenB |
| Gms0368 | 462 | 29 | e-130 | SrfAC |
| Gms4064 | 234 | 32 | 6e-062 | DltA |

**Table S3** The homologues of FenD of FZB42 detected by scanning the local NCD-2 proteome in BioEdit.

| protein number | score | similarity | E-value | function description |
| --- | --- | --- | --- | --- |
| Gms1960 | 1719 | 38 | 0.0 | FenA |
| Gms0365 | 1715 | 37 | 0.0 | SrfAA |
| Gms1961 | 1706 | 39 | 0.0 | FenE |
| Gms0366 | 1481 | 35 | 0.0 | Surfactin synthase subunit 1 |
| Gms3368 | 1179 | 35 | 0.0 | DhbF |
| Gms1959 | 814 | 41 | 0.0 | FenB |
| Gms0368 | 739 | 38 | 0.0 | SrfAC |
| Gms1826 | 620 | 27 | e-178 | PKSJ |
| Gms1829 | 560 | 32 | e-160 | PKSN |
| Gms0367 | 519 | 40 | e-148 | Surfactin synthase subunit 2 |

**Table S4** Adenylation domain binding amino acids predicted by PRISM.

| Gms1961 | A domain A9 | Val | Ile | Leu | Val | Phe | Asp | Tyr | Glu | Ala | N5-hydroxy-Orn |
| --- | --- | --- | --- | --- | --- | --- | --- | --- | --- | --- | --- |
|  | score | 943.0 | 595.0 | 566.0 | 556.5 | 515.2 | 483.3 | 482.8 | 482.7 | 475.2 | 109.9 |
| Gms1959 | A domain A13 | Ile | Val | Val | Leu | Phe | Ala | Tyr | Leu | Glut | β-Phe |
|  | score | 819.1 | 645.3 | 535.3 | 508.4 | 469.7 | 430.9 | 424.1 | 421.7 | 402.9 | 109.1 |

Predicted by PRISM (http://grid.adapsyn.com/prism/). The scores represent the ability of the adenylation domain binding amino acids.
