# Supplementary figures and images for: Genome mining and UHPLC–QTOF–MS/MS to identify the potential antimicrobial compounds and determine the specificity of biosynthetic gene clusters in Bacillus subtilis NCD-2
Source: BMC Genomics. 2020 Nov 5;21:767. doi: 10.1186/s12864-020-07160-2 (PMC7643408; doi:10.1186/s12864-020-07160-2)

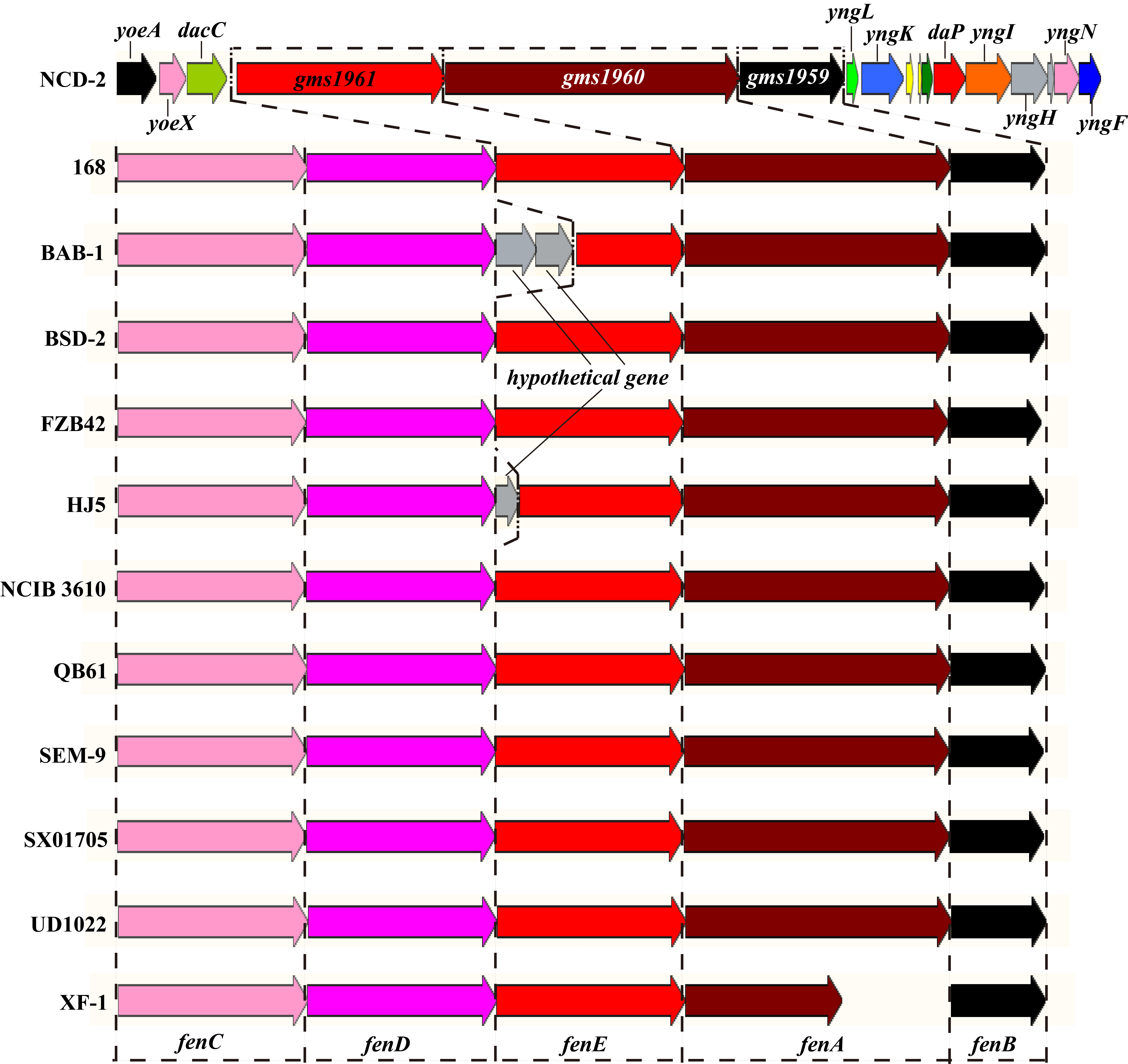

Supplement: Supplementary file 1 — Additional file 1 : Fig. S1. Fengycin biosynthetic gene clusters of different strains that have a close relation with NCD-2 or the model strains. Fig. S2. Surfactin biosynthetic gene clusters of different strains that have a close relation with NCD-2 or the model strains. Fig. S3. Elution of lipopeptides separated from the crude methanolic extract using an AKTA Purifier. Fig. S4. Primary structures of fengycins and surfactins. Fig. S5. Fengycin A of a β-OH FA with a chain length varying from C14 to C19 identified based on key product ions. Fig. S6. Fengycin B of a β-OH FA with a chain length varying from C12 to C19 identified based on key product ions. Fig. S7. Fengycin A2 of a β-OH FA with a chain length varying from C15-C18 identified based on key product ions. Fig. S8. Fengycin B2 of a β-OH FA with a chain length varying from C14-C18 identified based on key product ions. Fig. S9. Fengycin C of a β-OH FA with a chain length varying from C18-C20 identified based on key product ions. Fig. S10. Surfactin of a fatty acid with a chain length varying from C11-C15 identified based on key product ions. Fig. S11 Original, full-length gel images. Table S1. All B. subtilis strains with the assembly level of chromosome and their RefSeq assembly accessions. Table S2. Homologues of FenC of FZB42 detected by scanning the local NCD-2 proteome in BioEdit. Table S3. Homologues of FenD of FZB42 detected by scanning the local NCD-2 proteome in BioEdit. Table S4. Adenylation domain binding amino acids predicted by PRISM. [file 12864_2020_7160_MOESM1_ESM.zip › Fig.S1 .jpg]

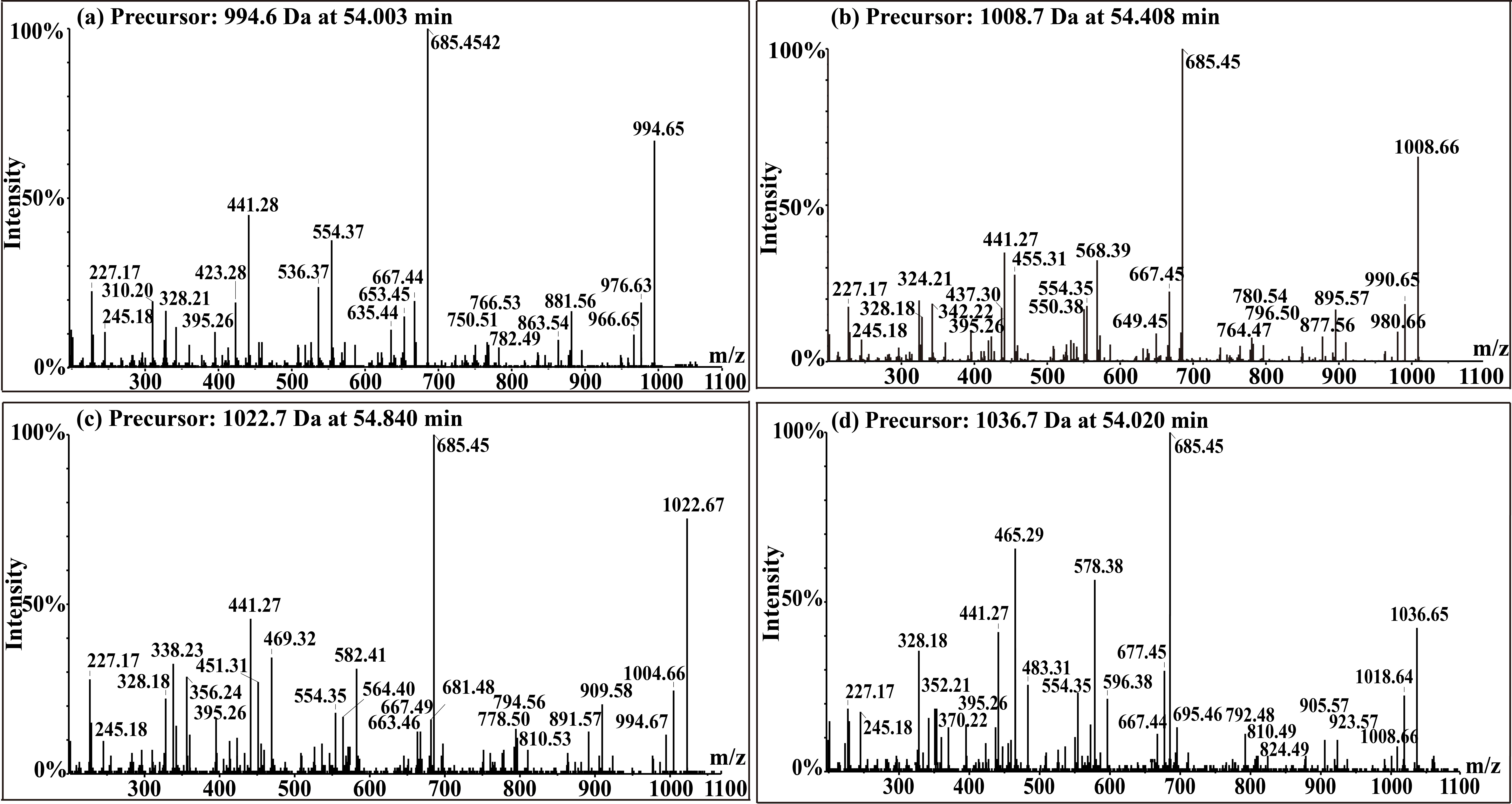

Supplement: Supplementary file 1 — Additional file 1 : Fig. S1. Fengycin biosynthetic gene clusters of different strains that have a close relation with NCD-2 or the model strains. Fig. S2. Surfactin biosynthetic gene clusters of different strains that have a close relation with NCD-2 or the model strains. Fig. S3. Elution of lipopeptides separated from the crude methanolic extract using an AKTA Purifier. Fig. S4. Primary structures of fengycins and surfactins. Fig. S5. Fengycin A of a β-OH FA with a chain length varying from C14 to C19 identified based on key product ions. Fig. S6. Fengycin B of a β-OH FA with a chain length varying from C12 to C19 identified based on key product ions. Fig. S7. Fengycin A2 of a β-OH FA with a chain length varying from C15-C18 identified based on key product ions. Fig. S8. Fengycin B2 of a β-OH FA with a chain length varying from C14-C18 identified based on key product ions. Fig. S9. Fengycin C of a β-OH FA with a chain length varying from C18-C20 identified based on key product ions. Fig. S10. Surfactin of a fatty acid with a chain length varying from C11-C15 identified based on key product ions. Fig. S11 Original, full-length gel images. Table S1. All B. subtilis strains with the assembly level of chromosome and their RefSeq assembly accessions. Table S2. Homologues of FenC of FZB42 detected by scanning the local NCD-2 proteome in BioEdit. Table S3. Homologues of FenD of FZB42 detected by scanning the local NCD-2 proteome in BioEdit. Table S4. Adenylation domain binding amino acids predicted by PRISM. [file 12864_2020_7160_MOESM1_ESM.zip › Fig.S10.jpg]

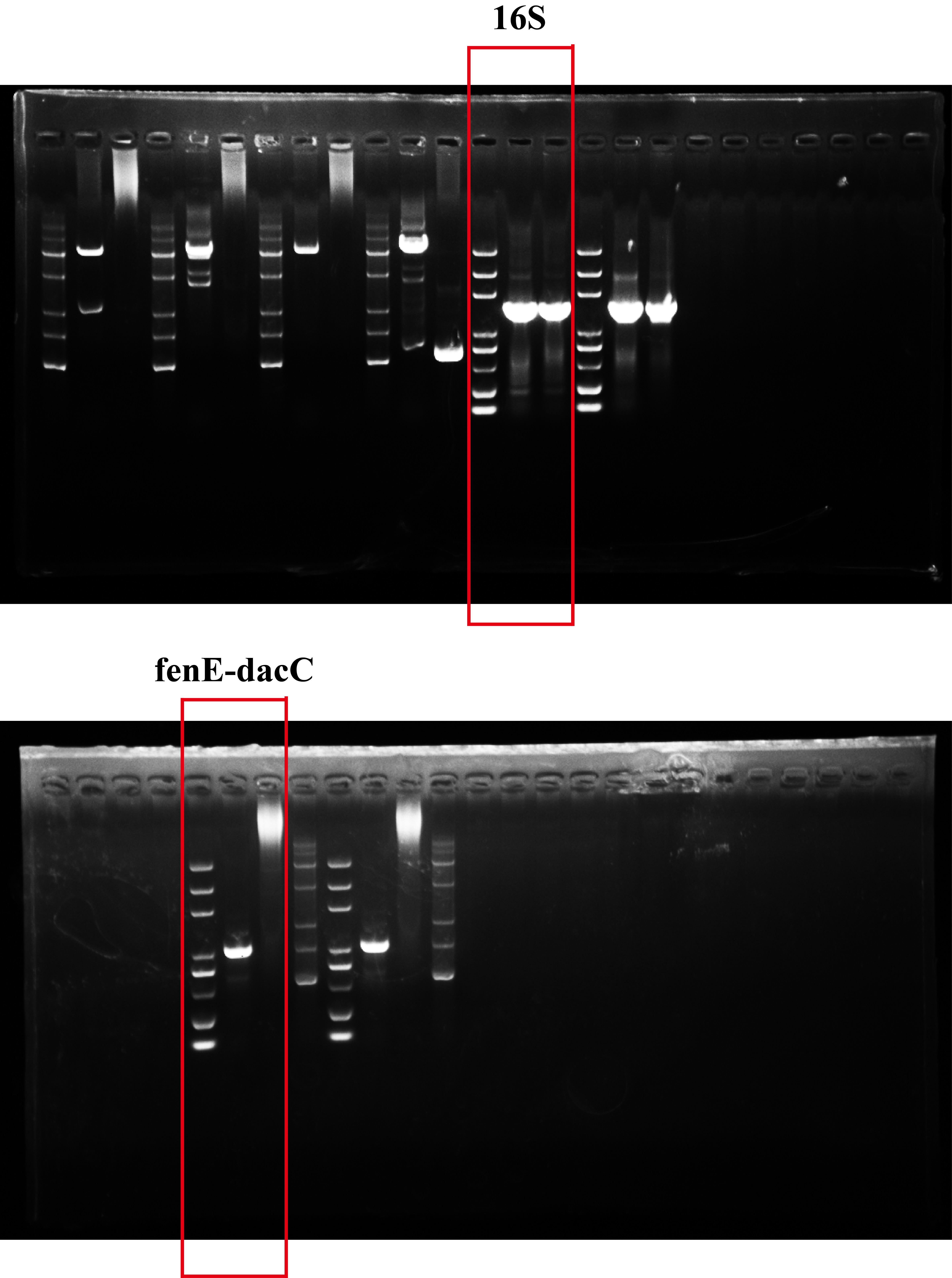

Supplement: Supplementary file 1 — Additional file 1 : Fig. S1. Fengycin biosynthetic gene clusters of different strains that have a close relation with NCD-2 or the model strains. Fig. S2. Surfactin biosynthetic gene clusters of different strains that have a close relation with NCD-2 or the model strains. Fig. S3. Elution of lipopeptides separated from the crude methanolic extract using an AKTA Purifier. Fig. S4. Primary structures of fengycins and surfactins. Fig. S5. Fengycin A of a β-OH FA with a chain length varying from C14 to C19 identified based on key product ions. Fig. S6. Fengycin B of a β-OH FA with a chain length varying from C12 to C19 identified based on key product ions. Fig. S7. Fengycin A2 of a β-OH FA with a chain length varying from C15-C18 identified based on key product ions. Fig. S8. Fengycin B2 of a β-OH FA with a chain length varying from C14-C18 identified based on key product ions. Fig. S9. Fengycin C of a β-OH FA with a chain length varying from C18-C20 identified based on key product ions. Fig. S10. Surfactin of a fatty acid with a chain length varying from C11-C15 identified based on key product ions. Fig. S11 Original, full-length gel images. Table S1. All B. subtilis strains with the assembly level of chromosome and their RefSeq assembly accessions. Table S2. Homologues of FenC of FZB42 detected by scanning the local NCD-2 proteome in BioEdit. Table S3. Homologues of FenD of FZB42 detected by scanning the local NCD-2 proteome in BioEdit. Table S4. Adenylation domain binding amino acids predicted by PRISM. [file 12864_2020_7160_MOESM1_ESM.zip › Fig.S11 original image.jpg]

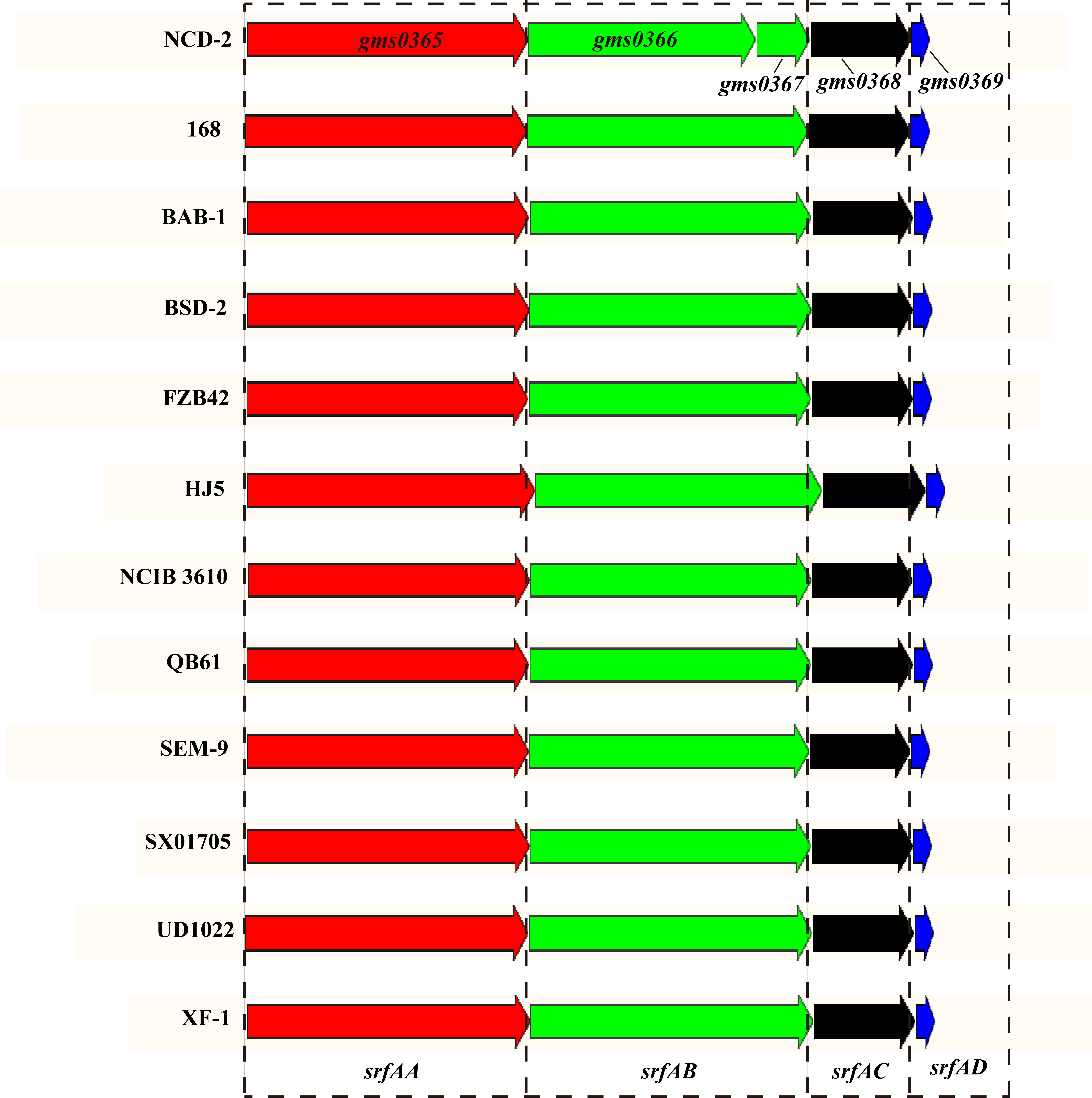

Supplement: Supplementary file 1 — Additional file 1 : Fig. S1. Fengycin biosynthetic gene clusters of different strains that have a close relation with NCD-2 or the model strains. Fig. S2. Surfactin biosynthetic gene clusters of different strains that have a close relation with NCD-2 or the model strains. Fig. S3. Elution of lipopeptides separated from the crude methanolic extract using an AKTA Purifier. Fig. S4. Primary structures of fengycins and surfactins. Fig. S5. Fengycin A of a β-OH FA with a chain length varying from C14 to C19 identified based on key product ions. Fig. S6. Fengycin B of a β-OH FA with a chain length varying from C12 to C19 identified based on key product ions. Fig. S7. Fengycin A2 of a β-OH FA with a chain length varying from C15-C18 identified based on key product ions. Fig. S8. Fengycin B2 of a β-OH FA with a chain length varying from C14-C18 identified based on key product ions. Fig. S9. Fengycin C of a β-OH FA with a chain length varying from C18-C20 identified based on key product ions. Fig. S10. Surfactin of a fatty acid with a chain length varying from C11-C15 identified based on key product ions. Fig. S11 Original, full-length gel images. Table S1. All B. subtilis strains with the assembly level of chromosome and their RefSeq assembly accessions. Table S2. Homologues of FenC of FZB42 detected by scanning the local NCD-2 proteome in BioEdit. Table S3. Homologues of FenD of FZB42 detected by scanning the local NCD-2 proteome in BioEdit. Table S4. Adenylation domain binding amino acids predicted by PRISM. [file 12864_2020_7160_MOESM1_ESM.zip › Fig.S2.jpg]

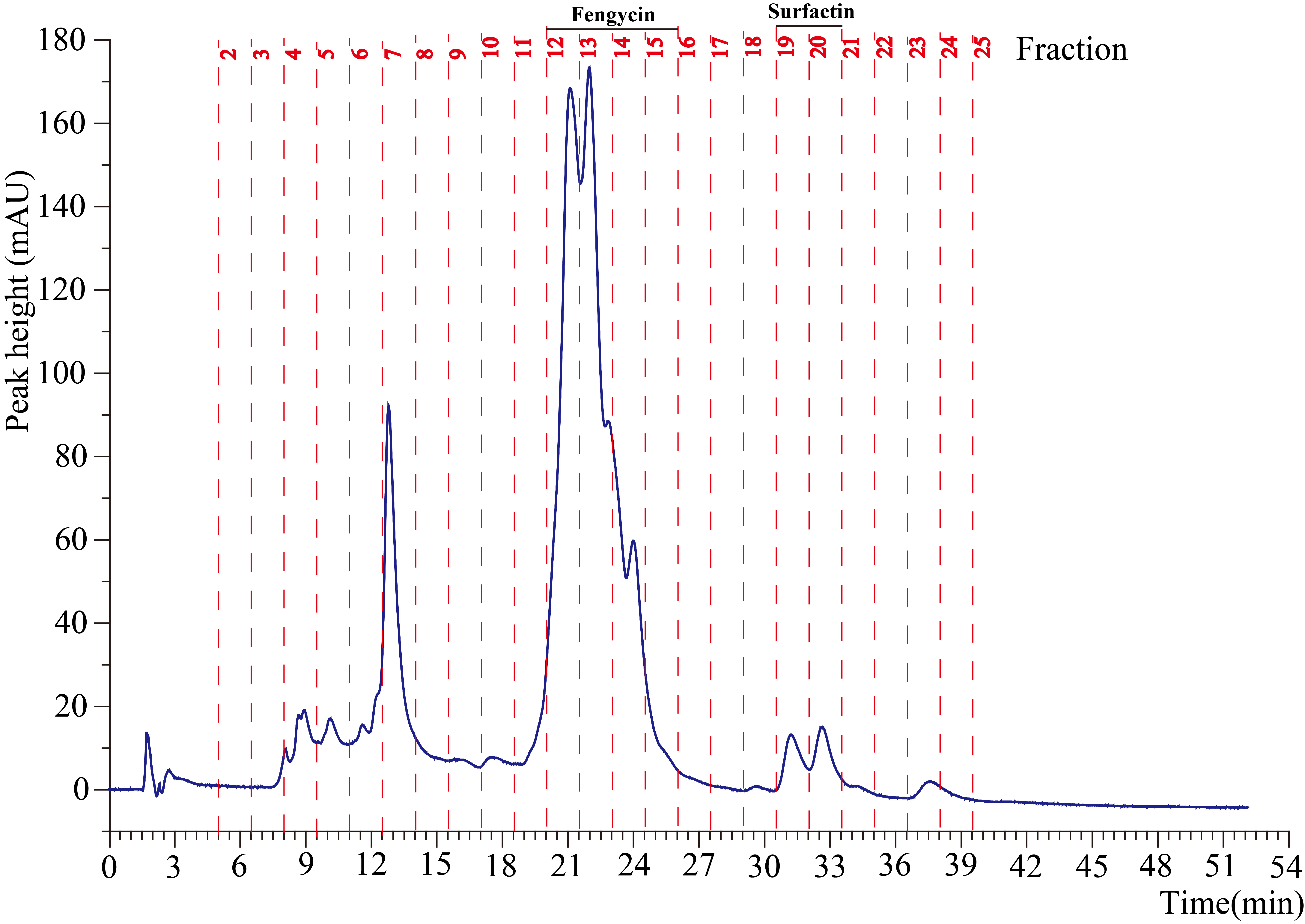

Supplement: Supplementary file 1 — Additional file 1 : Fig. S1. Fengycin biosynthetic gene clusters of different strains that have a close relation with NCD-2 or the model strains. Fig. S2. Surfactin biosynthetic gene clusters of different strains that have a close relation with NCD-2 or the model strains. Fig. S3. Elution of lipopeptides separated from the crude methanolic extract using an AKTA Purifier. Fig. S4. Primary structures of fengycins and surfactins. Fig. S5. Fengycin A of a β-OH FA with a chain length varying from C14 to C19 identified based on key product ions. Fig. S6. Fengycin B of a β-OH FA with a chain length varying from C12 to C19 identified based on key product ions. Fig. S7. Fengycin A2 of a β-OH FA with a chain length varying from C15-C18 identified based on key product ions. Fig. S8. Fengycin B2 of a β-OH FA with a chain length varying from C14-C18 identified based on key product ions. Fig. S9. Fengycin C of a β-OH FA with a chain length varying from C18-C20 identified based on key product ions. Fig. S10. Surfactin of a fatty acid with a chain length varying from C11-C15 identified based on key product ions. Fig. S11 Original, full-length gel images. Table S1. All B. subtilis strains with the assembly level of chromosome and their RefSeq assembly accessions. Table S2. Homologues of FenC of FZB42 detected by scanning the local NCD-2 proteome in BioEdit. Table S3. Homologues of FenD of FZB42 detected by scanning the local NCD-2 proteome in BioEdit. Table S4. Adenylation domain binding amino acids predicted by PRISM. [file 12864_2020_7160_MOESM1_ESM.zip › Fig.S3.jpg]

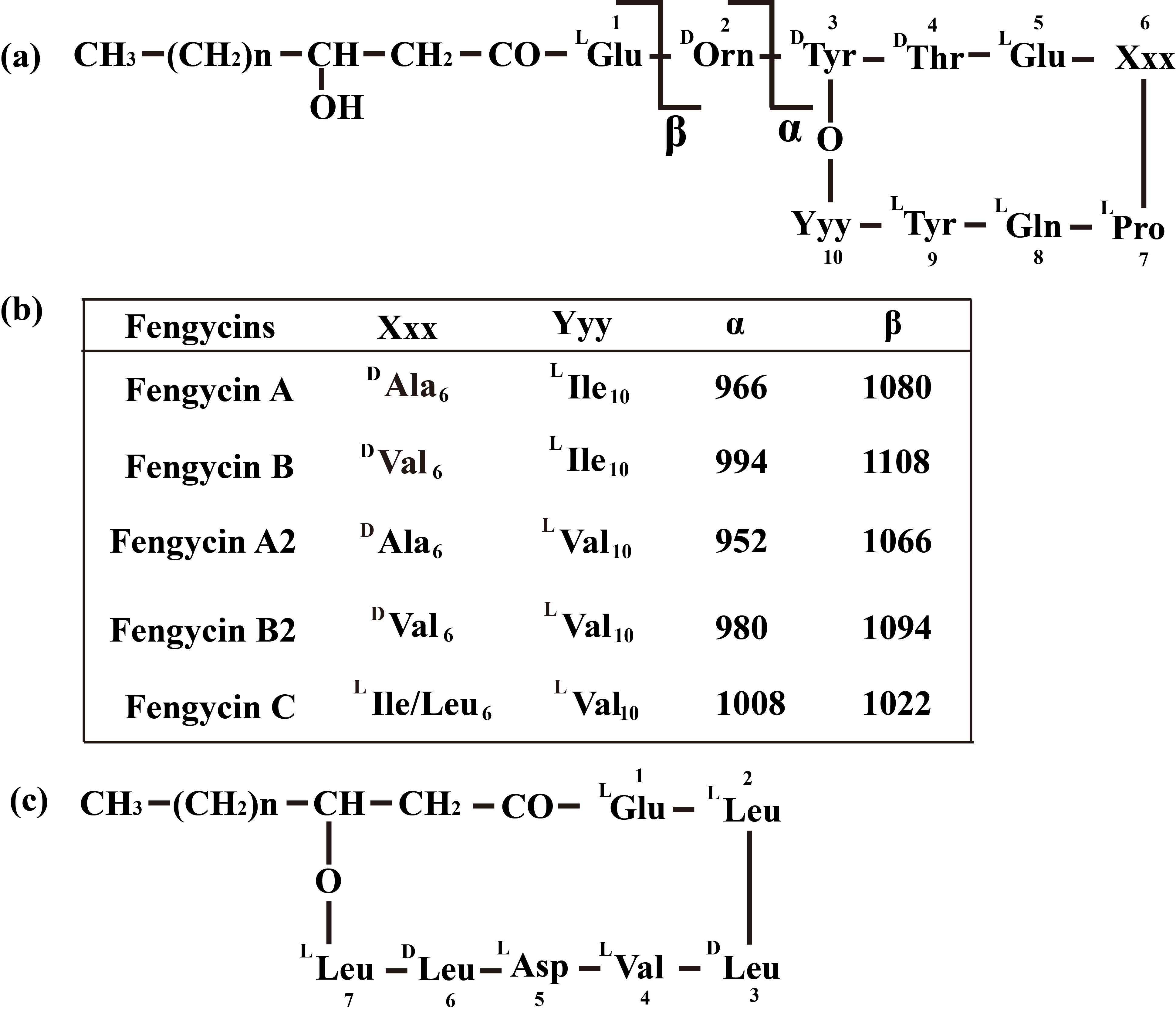

Supplement: Supplementary file 1 — Additional file 1 : Fig. S1. Fengycin biosynthetic gene clusters of different strains that have a close relation with NCD-2 or the model strains. Fig. S2. Surfactin biosynthetic gene clusters of different strains that have a close relation with NCD-2 or the model strains. Fig. S3. Elution of lipopeptides separated from the crude methanolic extract using an AKTA Purifier. Fig. S4. Primary structures of fengycins and surfactins. Fig. S5. Fengycin A of a β-OH FA with a chain length varying from C14 to C19 identified based on key product ions. Fig. S6. Fengycin B of a β-OH FA with a chain length varying from C12 to C19 identified based on key product ions. Fig. S7. Fengycin A2 of a β-OH FA with a chain length varying from C15-C18 identified based on key product ions. Fig. S8. Fengycin B2 of a β-OH FA with a chain length varying from C14-C18 identified based on key product ions. Fig. S9. Fengycin C of a β-OH FA with a chain length varying from C18-C20 identified based on key product ions. Fig. S10. Surfactin of a fatty acid with a chain length varying from C11-C15 identified based on key product ions. Fig. S11 Original, full-length gel images. Table S1. All B. subtilis strains with the assembly level of chromosome and their RefSeq assembly accessions. Table S2. Homologues of FenC of FZB42 detected by scanning the local NCD-2 proteome in BioEdit. Table S3. Homologues of FenD of FZB42 detected by scanning the local NCD-2 proteome in BioEdit. Table S4. Adenylation domain binding amino acids predicted by PRISM. [file 12864_2020_7160_MOESM1_ESM.zip › Fig.S4.jpg]

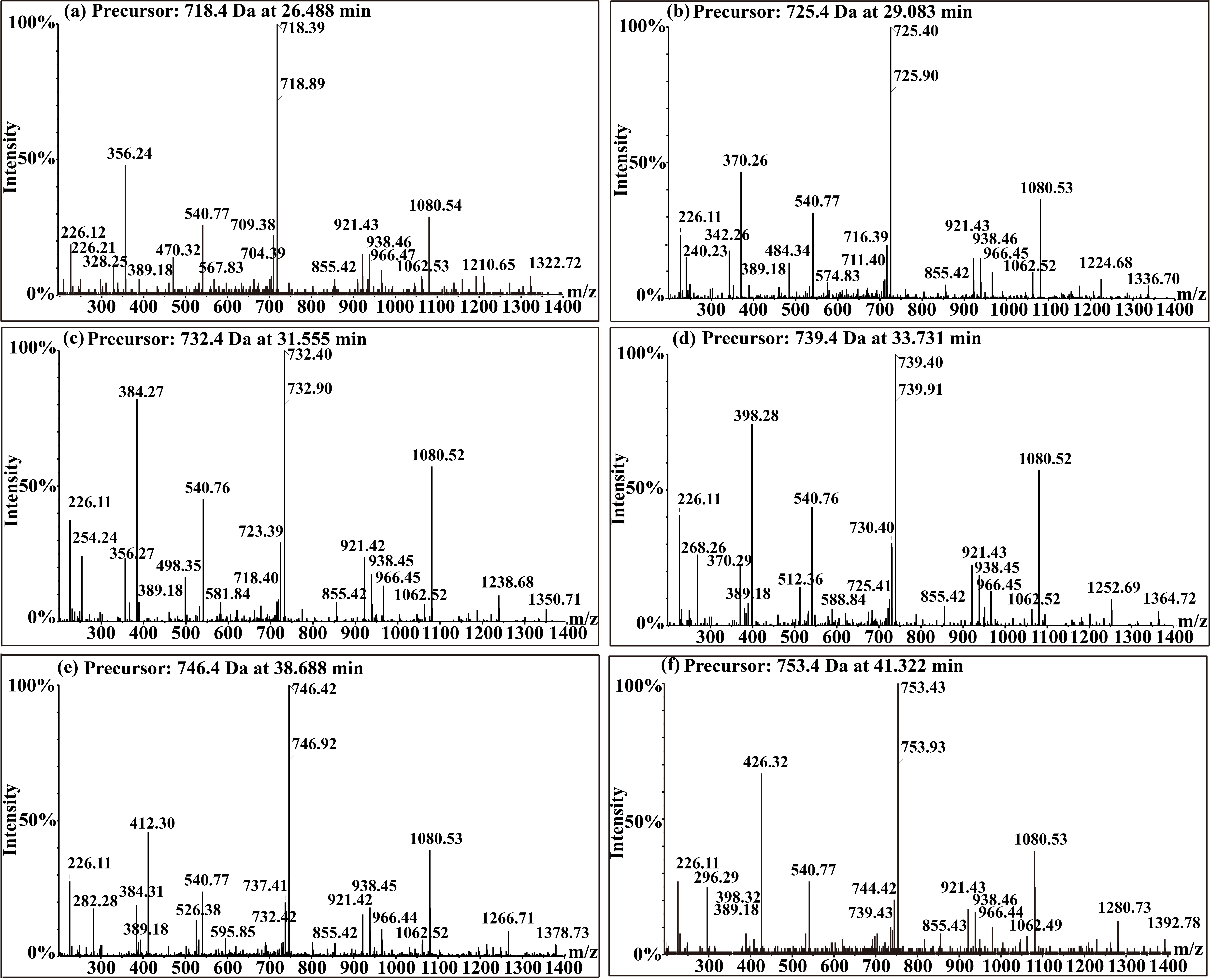

Supplement: Supplementary file 1 — Additional file 1 : Fig. S1. Fengycin biosynthetic gene clusters of different strains that have a close relation with NCD-2 or the model strains. Fig. S2. Surfactin biosynthetic gene clusters of different strains that have a close relation with NCD-2 or the model strains. Fig. S3. Elution of lipopeptides separated from the crude methanolic extract using an AKTA Purifier. Fig. S4. Primary structures of fengycins and surfactins. Fig. S5. Fengycin A of a β-OH FA with a chain length varying from C14 to C19 identified based on key product ions. Fig. S6. Fengycin B of a β-OH FA with a chain length varying from C12 to C19 identified based on key product ions. Fig. S7. Fengycin A2 of a β-OH FA with a chain length varying from C15-C18 identified based on key product ions. Fig. S8. Fengycin B2 of a β-OH FA with a chain length varying from C14-C18 identified based on key product ions. Fig. S9. Fengycin C of a β-OH FA with a chain length varying from C18-C20 identified based on key product ions. Fig. S10. Surfactin of a fatty acid with a chain length varying from C11-C15 identified based on key product ions. Fig. S11 Original, full-length gel images. Table S1. All B. subtilis strains with the assembly level of chromosome and their RefSeq assembly accessions. Table S2. Homologues of FenC of FZB42 detected by scanning the local NCD-2 proteome in BioEdit. Table S3. Homologues of FenD of FZB42 detected by scanning the local NCD-2 proteome in BioEdit. Table S4. Adenylation domain binding amino acids predicted by PRISM. [file 12864_2020_7160_MOESM1_ESM.zip › Fig.S5.jpg]

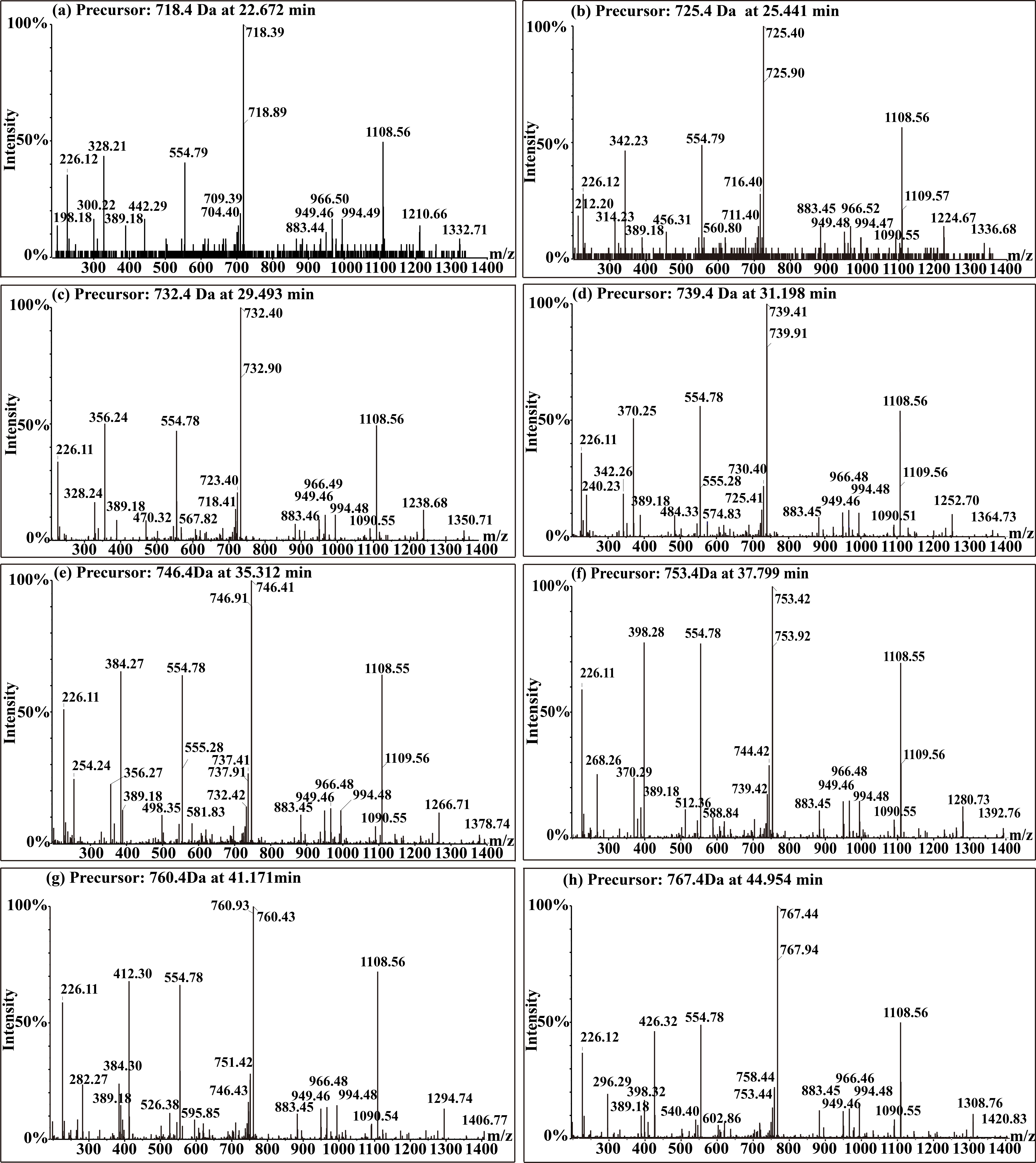

Supplement: Supplementary file 1 — Additional file 1 : Fig. S1. Fengycin biosynthetic gene clusters of different strains that have a close relation with NCD-2 or the model strains. Fig. S2. Surfactin biosynthetic gene clusters of different strains that have a close relation with NCD-2 or the model strains. Fig. S3. Elution of lipopeptides separated from the crude methanolic extract using an AKTA Purifier. Fig. S4. Primary structures of fengycins and surfactins. Fig. S5. Fengycin A of a β-OH FA with a chain length varying from C14 to C19 identified based on key product ions. Fig. S6. Fengycin B of a β-OH FA with a chain length varying from C12 to C19 identified based on key product ions. Fig. S7. Fengycin A2 of a β-OH FA with a chain length varying from C15-C18 identified based on key product ions. Fig. S8. Fengycin B2 of a β-OH FA with a chain length varying from C14-C18 identified based on key product ions. Fig. S9. Fengycin C of a β-OH FA with a chain length varying from C18-C20 identified based on key product ions. Fig. S10. Surfactin of a fatty acid with a chain length varying from C11-C15 identified based on key product ions. Fig. S11 Original, full-length gel images. Table S1. All B. subtilis strains with the assembly level of chromosome and their RefSeq assembly accessions. Table S2. Homologues of FenC of FZB42 detected by scanning the local NCD-2 proteome in BioEdit. Table S3. Homologues of FenD of FZB42 detected by scanning the local NCD-2 proteome in BioEdit. Table S4. Adenylation domain binding amino acids predicted by PRISM. [file 12864_2020_7160_MOESM1_ESM.zip › Fig.S6.jpg]

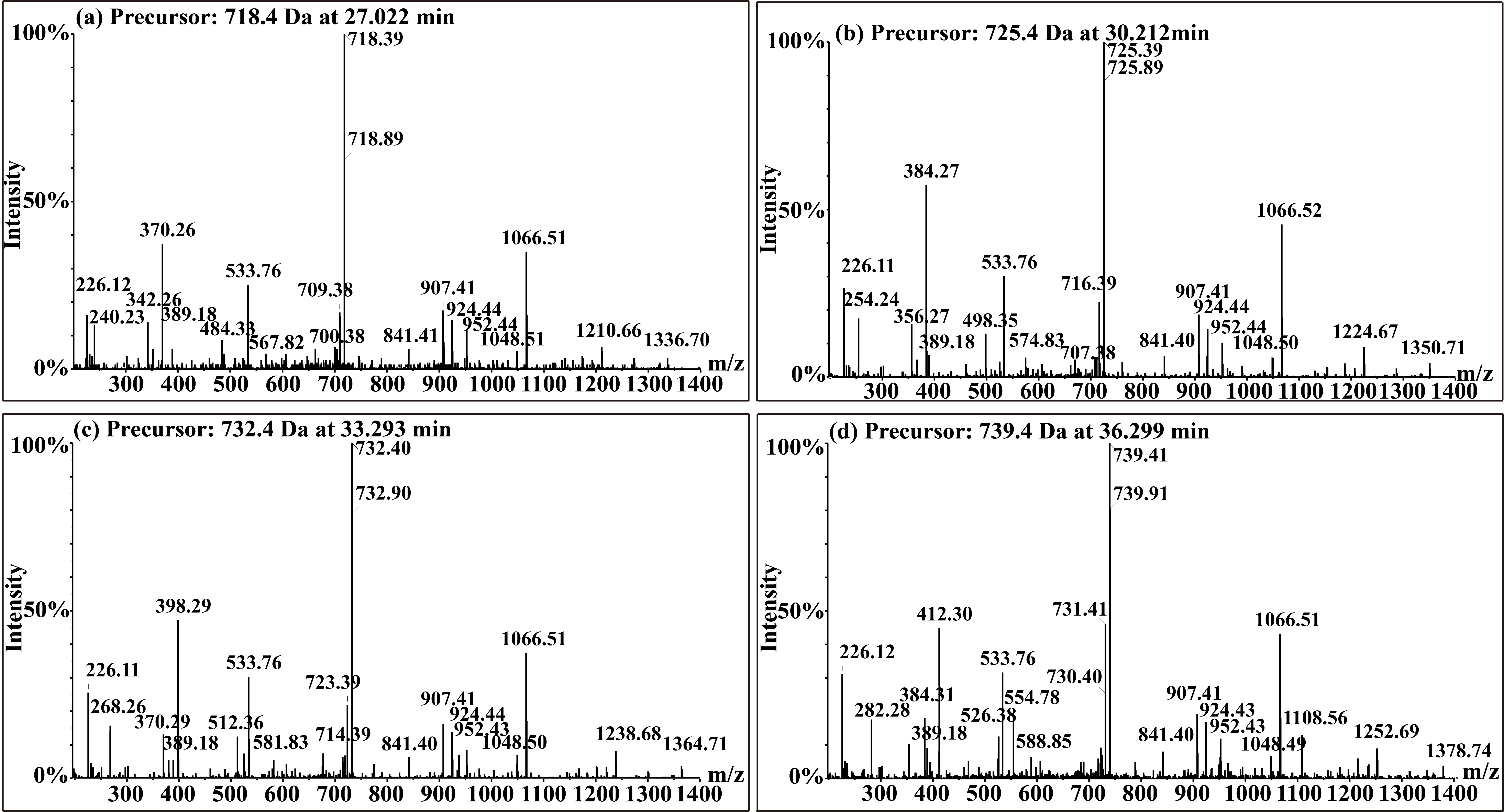

Supplement: Supplementary file 1 — Additional file 1 : Fig. S1. Fengycin biosynthetic gene clusters of different strains that have a close relation with NCD-2 or the model strains. Fig. S2. Surfactin biosynthetic gene clusters of different strains that have a close relation with NCD-2 or the model strains. Fig. S3. Elution of lipopeptides separated from the crude methanolic extract using an AKTA Purifier. Fig. S4. Primary structures of fengycins and surfactins. Fig. S5. Fengycin A of a β-OH FA with a chain length varying from C14 to C19 identified based on key product ions. Fig. S6. Fengycin B of a β-OH FA with a chain length varying from C12 to C19 identified based on key product ions. Fig. S7. Fengycin A2 of a β-OH FA with a chain length varying from C15-C18 identified based on key product ions. Fig. S8. Fengycin B2 of a β-OH FA with a chain length varying from C14-C18 identified based on key product ions. Fig. S9. Fengycin C of a β-OH FA with a chain length varying from C18-C20 identified based on key product ions. Fig. S10. Surfactin of a fatty acid with a chain length varying from C11-C15 identified based on key product ions. Fig. S11 Original, full-length gel images. Table S1. All B. subtilis strains with the assembly level of chromosome and their RefSeq assembly accessions. Table S2. Homologues of FenC of FZB42 detected by scanning the local NCD-2 proteome in BioEdit. Table S3. Homologues of FenD of FZB42 detected by scanning the local NCD-2 proteome in BioEdit. Table S4. Adenylation domain binding amino acids predicted by PRISM. [file 12864_2020_7160_MOESM1_ESM.zip › Fig.S7.jpg]

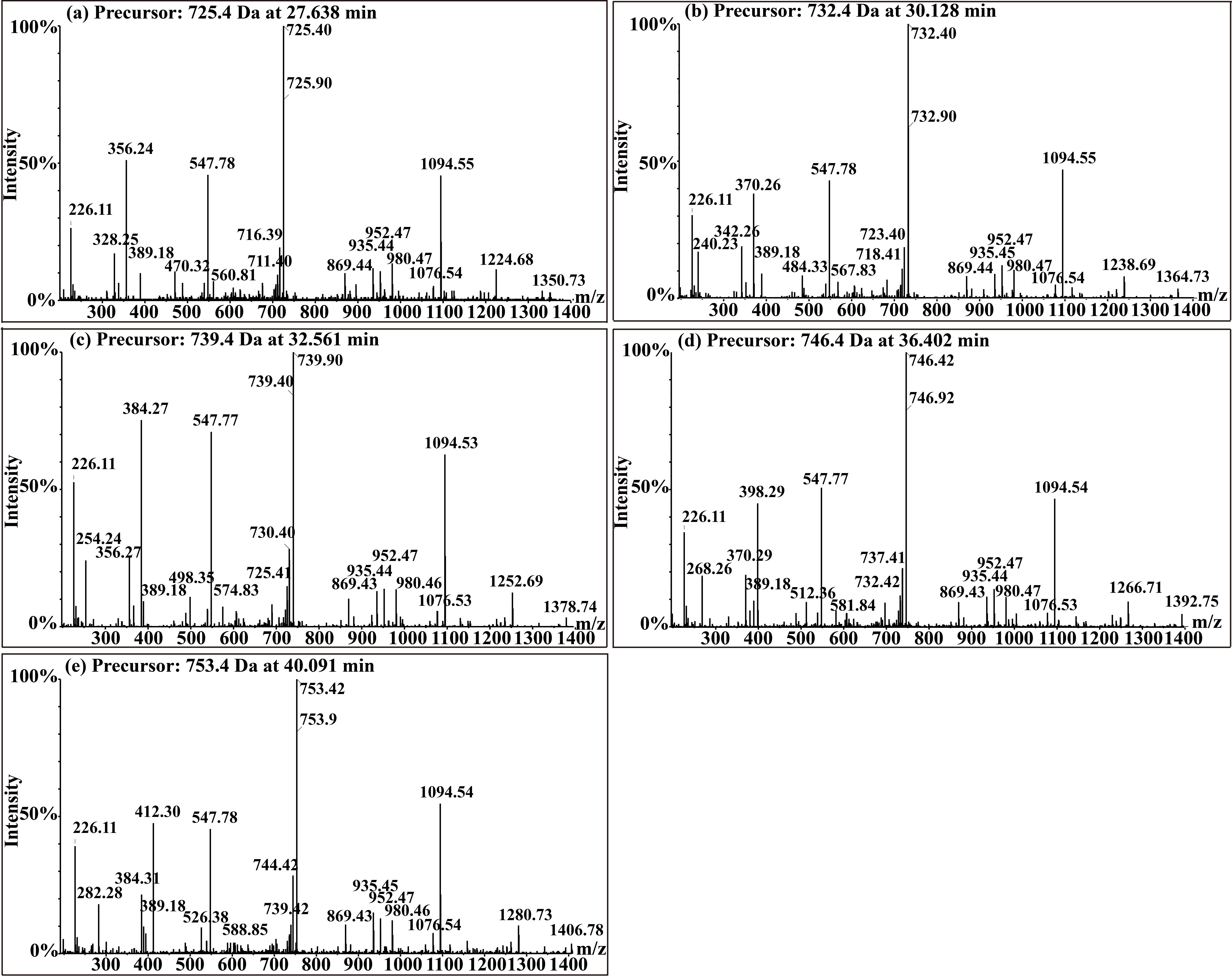

Supplement: Supplementary file 1 — Additional file 1 : Fig. S1. Fengycin biosynthetic gene clusters of different strains that have a close relation with NCD-2 or the model strains. Fig. S2. Surfactin biosynthetic gene clusters of different strains that have a close relation with NCD-2 or the model strains. Fig. S3. Elution of lipopeptides separated from the crude methanolic extract using an AKTA Purifier. Fig. S4. Primary structures of fengycins and surfactins. Fig. S5. Fengycin A of a β-OH FA with a chain length varying from C14 to C19 identified based on key product ions. Fig. S6. Fengycin B of a β-OH FA with a chain length varying from C12 to C19 identified based on key product ions. Fig. S7. Fengycin A2 of a β-OH FA with a chain length varying from C15-C18 identified based on key product ions. Fig. S8. Fengycin B2 of a β-OH FA with a chain length varying from C14-C18 identified based on key product ions. Fig. S9. Fengycin C of a β-OH FA with a chain length varying from C18-C20 identified based on key product ions. Fig. S10. Surfactin of a fatty acid with a chain length varying from C11-C15 identified based on key product ions. Fig. S11 Original, full-length gel images. Table S1. All B. subtilis strains with the assembly level of chromosome and their RefSeq assembly accessions. Table S2. Homologues of FenC of FZB42 detected by scanning the local NCD-2 proteome in BioEdit. Table S3. Homologues of FenD of FZB42 detected by scanning the local NCD-2 proteome in BioEdit. Table S4. Adenylation domain binding amino acids predicted by PRISM. [file 12864_2020_7160_MOESM1_ESM.zip › Fig.S8.jpg]

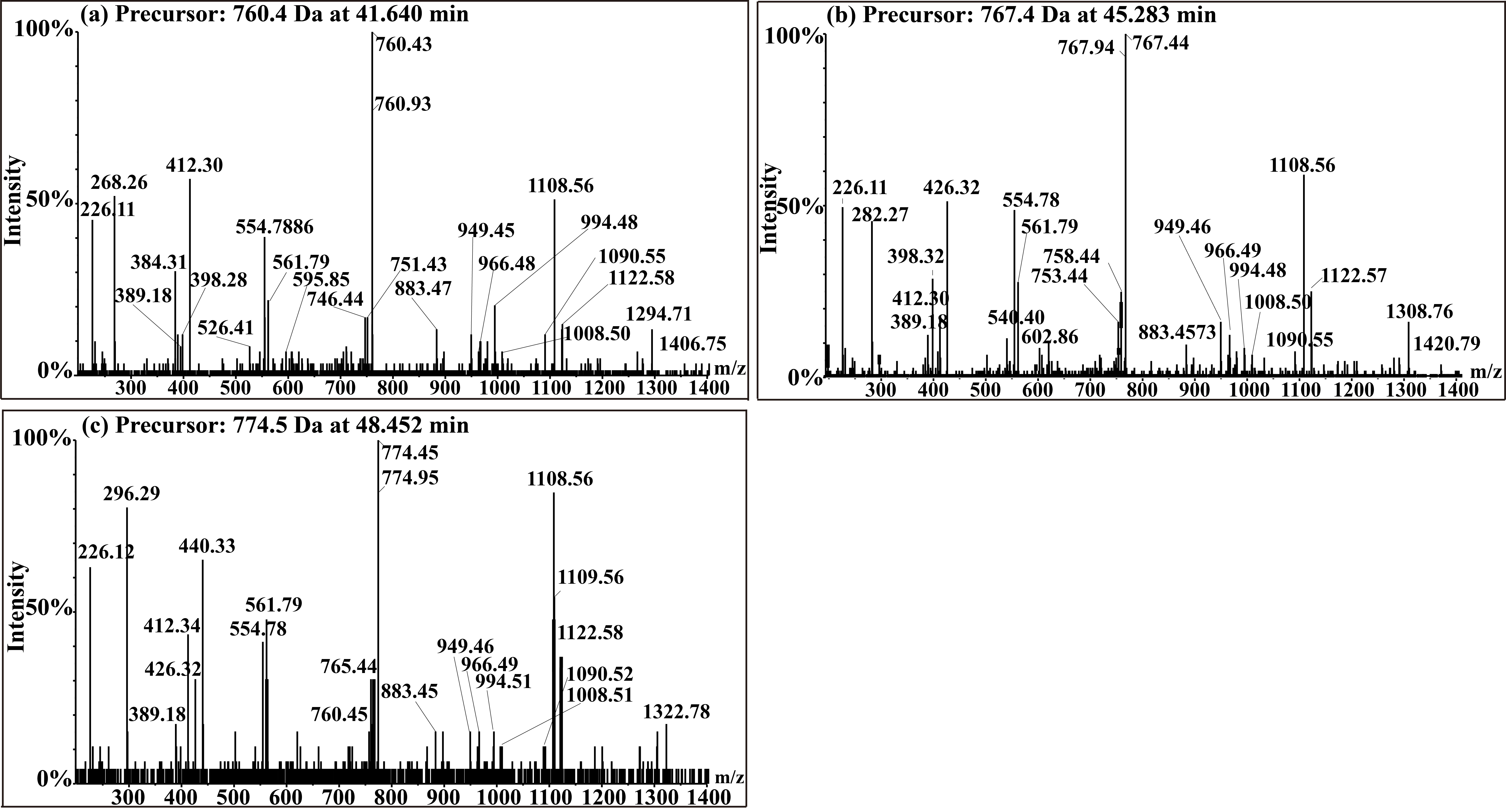

Supplement: Supplementary file 1 — Additional file 1 : Fig. S1. Fengycin biosynthetic gene clusters of different strains that have a close relation with NCD-2 or the model strains. Fig. S2. Surfactin biosynthetic gene clusters of different strains that have a close relation with NCD-2 or the model strains. Fig. S3. Elution of lipopeptides separated from the crude methanolic extract using an AKTA Purifier. Fig. S4. Primary structures of fengycins and surfactins. Fig. S5. Fengycin A of a β-OH FA with a chain length varying from C14 to C19 identified based on key product ions. Fig. S6. Fengycin B of a β-OH FA with a chain length varying from C12 to C19 identified based on key product ions. Fig. S7. Fengycin A2 of a β-OH FA with a chain length varying from C15-C18 identified based on key product ions. Fig. S8. Fengycin B2 of a β-OH FA with a chain length varying from C14-C18 identified based on key product ions. Fig. S9. Fengycin C of a β-OH FA with a chain length varying from C18-C20 identified based on key product ions. Fig. S10. Surfactin of a fatty acid with a chain length varying from C11-C15 identified based on key product ions. Fig. S11 Original, full-length gel images. Table S1. All B. subtilis strains with the assembly level of chromosome and their RefSeq assembly accessions. Table S2. Homologues of FenC of FZB42 detected by scanning the local NCD-2 proteome in BioEdit. Table S3. Homologues of FenD of FZB42 detected by scanning the local NCD-2 proteome in BioEdit. Table S4. Adenylation domain binding amino acids predicted by PRISM. [file 12864_2020_7160_MOESM1_ESM.zip › Fig.S9.jpg]
